# Genetic Association of Pulmonary Surfactant Protein Genes, SFTPA1, SFTPA2, SFTPB, SFTPC, and SFTPD With Cystic Fibrosis

**DOI:** 10.3389/fimmu.2018.02256

**Published:** 2018-10-02

**Authors:** Zhenwu Lin, Nithyananda Thorenoor, Rongling Wu, Susan L. DiAngelo, Meixia Ye, Neal J. Thomas, Xiaojie Liao, Tony R. Lin, Stuart Warren, Joanna Floros

**Affiliations:** ^1^Department of Radiology, University of Pennsylvania Perelman School of Medicine, Philadelphia, PA, United States; ^2^Department of Pediatrics, Center for Host Defense, Inflammation, and Lung Disease (CHILD) Research, Pennsylvania State University, Hershey, PA, United States; ^3^Public Health Science, College of Medicine, Pennsylvania State University, Hershey, PA, United States; ^4^Center for Computational Biology, College of Biological Sciences and Technology, Beijing Forestry University, Beijing, China; ^5^Obstetrics and Gynecology, Pennsylvania State University College of Medicine, Hershey, PA, United States

**Keywords:** surfactant protein, SP-A, SP-B, SP-C, SP-D, cystic fibrosis

## Abstract

Surfactant proteins (SP) are involved in surfactant function and innate immunity in the human lung. Both lung function and innate immunity are altered in CF, and altered SP levels and genetic association are observed in Cystic Fibrosis (CF). We hypothesized that single nucleotide polymorphisms (SNPs) within the SP genes associate with CF or severity subgroups, either through single SNP or via SNP-SNP interactions between two SNPs of a given gene (intragenic) and/or between two genes (intergenic). We genotyped a total of 17 SP SNPs from 72 case-trio pedigree (SFTPA1 (5), SFTPA2 (4), SFTPB (4), SFTPC (2), and SFTPD (2)), and identified SP SNP associations by applying quantitative genetic principles. The results showed (a) Two SNPs, SFTPB rs7316 (*p* = 0.0083) and SFTPC rs1124 (*p* = 0.0154), each associated with CF. (b) Three intragenic SNP-SNP interactions, SFTPB (rs2077079, rs3024798), and SFTPA1 (rs1136451, rs1059057 and rs4253527), associated with CF. (c) A total of 34 intergenic SNP-SNP interactions among the 4 SP genes to be associated with CF. (d) No SNP-SNP interaction was observed between SFTPA1 or SFTPA2 and SFTPD. (e) Equal number of SNP-SNP interactions were observed between SFTPB and SFTPA1/SFTPA2 (*n* = 7) and SP-B and SFTPD (*n* = 7). (f) SFTPC exhibited significant SNP-SNP interactions with SFTPA1/SFTPA2 (*n* = 11), SFTPB (*n* = 4) and SFTPD (*n* = 3). (g) A single SFTPB SNP was associated with mild CF after Bonferroni correction, and several intergenic interactions that are associated (*p* < 0.01) with either mild or moderate/severe CF were observed. These collectively indicate that complex SNP-SNP interactions of the SP genes may contribute to the pulmonary disease in CF patients. We speculate that SPs may serve as modifiers for the varied progression of pulmonary disease in CF and/or its severity.

## Introduction

Cystic fibrosis (CF) is an autosomal multi-organ recessive inherited disease. Mutations in the cystic fibrosis transmembrane conductance regulator (CFTR) protein are key in CF pathogenesis (1). CFTR is activated via cAMP through β_2_ adrenoceptor stimulation; coding sequence polymorphisms in the CFTR result in CF (2). CFTR functions as a chloride channel on the surface of airway epithelial cells. In patients with CF, loss of CFTR channel function at the cell surface results in impermeability and increased sodium absorption (3, 4). Depletion of the airway surface liquid causes reduced mucus clearance resulting in bacterial colonization, recurrent infections, chronic inflammation, and irreversible damage to the airway epithelium.

Pulmonary function deterioration is one of the primary complications of CF and pulmonary surfactant is essential for normal lung function. Pulmonary surfactant, a surface-active lipoprotein complex, is composed of 90% lipids and 10% surfactant proteins. The latter includes plasma proteins and surfactant proteins SP-A1, SP-A2, SP-B, and SP-C. The surfactant proteins comprise a hydrophobic group of proteins (SP-B and SP-C) and a hydrophilic group of proteins (SP-A1 and SP-A2). The SP-D, although co-isolates with surfactant, is not an integral part of the surfactant complex, but it is grouped with SP-A1/A2 based on its structural similarity and function (5, 6). Pulmonary surfactant is synthesized and secreted by the alveolar epithelial Type II cells of the lung and maintains the stability of the pulmonary tissue by reducing the surface tension of fluids that coat the lung. Broadly speaking the hydrophobic surfactant proteins (SP-B and SP-C) are primarily involved in surface properties of surfactant and are important for normal lung function (7); the hydrophilic proteins (SP-A1, SP-A2, and SP-D) are primarily involved in host defense (6, 8, 9), although SP-A1 and SP-A2 exhibit differential effects on the surfactant structural reorganization (10), on the organization of phospholipid monolayers containing SP-B (11), and lung mechanics (12). Moreover, lipid-mediated interactions of SP-A/SP-B may contribute to normal lung surfactant function (13).

Unlike in rodents that have a single gene encoding surfactant protein A (SP-A), in humans SP-A consists of SP-A1 and SP-A2 encoded by SFTPA1 and SFTPA2, respectively; each gene has been identified with several genetic variants (14), and these have been shown to associate with several pulmonary diseases (15, 16). The human SP-D locus is linked to the SP-A locus and is located proximal to the centromere at approximately 80–100 kb from the SFTPA2 gene (14). Genetic associations between SFTPD SNPs and lung disease have also been identified (17, 18). Although SP-A1, SP-A2, and SP-D are molecules of the innate immunity of the lung, there may be differences in the mechanisms via which host defense is achieved (19, 20). Surfactant proteins SP-B and SP-C play a key role in lung function in normal lung. SP-B is essential for life (21), and both SP-B and SP-C via their role in surfactant function may contribute to CF.

Lung surfactant proteins may contribute to the outcome in CF (22). Bronchoalveolar lavage levels of SP-A have been shown to be increased early in the course of the CF (23), but decrease as disease progresses. Lower levels are correlated with more inflammation and diminished lung function (22, 24–27). Although, the level of SP-B (encoded by SFTPB) was unchanged in BAL from CF patients (24, 28, 29), in CF patients with mild lung disease (30) SP-B was found to be increased, but SP-A did not change. No changes were observed in the levels of SP-C and SP-D, encoded by the SFTPC and SFTPD genes, respectively. However, increased levels of SP-D were observed in serum of CF patients (31).

Moreover, in CF patients with well-conserved lung function (26), SP-C was increased, SP-A was decreased but SP-B and SP-D were not changed. Recently, it was reported that in CF patients, complex forms of SP-A were associated with better lung function. This indicates that the structural organization of SP-A affects its functional activity and this is linked to disease severity (32). SP-A1, shown previously to form higher size oligomers compared to SP-A2 (33), was shown recently to affect more efficiently (than SP-A2) the structural organization of surfactant, which in turn may affect lung function (10). Furthermore, genetic associations of SFTPA1 and SFTPA2 with CF have been observed (34). Moreover, different size SP-D oligomers have been associated with functional differences in patients with chronic lung disease such as CF (35).

Because individuals with identical CFTR mutations may differ in their pulmonary disease, it has been postulated that other genetic factors (i.e. gene modifiers) as well as the microenvironment may contribute to the variable outcome of pulmonary disease (36–41). Surfactant proteins play an important role in surfactant function (7, 34, 42), pulmonary mechanics (12), and innate immunity (6, 8, 9). Furthermore, disruption of these functions can compromise normal lung health. Therefore, we postulated that the surfactant proteins contribute to the progression of the pulmonary disease in CF. We hypothesized that multiple genetic variants of the surfactant protein genes, SFTPA1, SFTPA2, SFTPB, SFTPC, and/or SFTPD, are associated with CF or disease severity subgroups (mild and moderate/severe) through single genetic variations within a gene, and through intragenic or intergenic interactions between variants of a single gene or variants of two different genes. Allele frequencies and linkage disequilibrium of these loci in races and ethnic groups has been previously studied (43). We further hypothesized that some of the associations are unique to patients with mild CF and moderate/severe CF. The observations made indicate that complex SNP-SNP interactions of the surfactant protein genes may contribute to the pulmonary disease in CF patients, and the SPs could serve as modifier genes in lung CF.

## Materials and methods

### Study samples

The patient samples were collected with informed and written consent from patients and/or parents under an approved protocol by the institutional review board from the Human Subject Protection Office of the Pennsylvania State University College of Medicine. The clinical data of the study samples are given in Supplementary Table 1 and summarized below.

Seventy-two pedigrees (family trees) were studied, of which, 43 pedigrees had one case with two parents, 21 pedigrees had one case with a single parent, five pedigrees had 2 cases with 1 or 2 parents, and 3 pedigrees had 1 or 2 cases in 3 generations. There were a total of 205 study samples in the 72 pedigrees and of these 79 were CF cases. Their ethnicity was as follows: 198-White, 7-Hispanic; 196-American, 6-Mexican, and 3- Unknown (Supplementary Table 1). A correction for ethnicity and sex was performed in the analysis but no correction was made for age. The lung function was assessed by standard spirometry, and for children, <5 years of age, assessment of disease severity was done by the clinical scoring of CXR by the Wisconsin Scoring System (44, 45). CF disease severity was classified as mild, moderate, and severe by lung function impairment based on percent predicted forced expired volume in 1 sec (FEV_1_). FEV_1_/FVC: mild = 70–89%; moderate = 40–69%; severe ≤ 40% (Cystic Fibrosis Foundation). The number of patients in the present study under this classification is as follows: severe in 4 cases, moderate in 11 cases, and mild in 64 cases.

### DNA isolation

Genomic DNAs were prepared from blood samples using QIAamp Blood kit following the manufacturer's instructions (Qiagen, Valencia, CA, United States).

### Selected genetic variations for this study

The target surfactant protein genes, SFTPA1, SFTPA2, SFTPB, SFTPC, and SFTPD, were selected based on gene function and association with lung diseases (especially with CF) from our findings and other published data as described above. A total of 17 genetic SNPs were selected. These SNPs were previously shown to associate with various lung diseases, be important in function or structure, or other: 5 SNPs from SFTPA1, rs1059047, rs1136450, rs1136451, rs1059057, and rs4253527; 4 SNPs from SFTPA2, rs1059046, rs17886395, rs1965707, and 1965708; 4 SNPs from SFTPB, rs7316, rs2077079, rs3024798, and rs1130866; 2 SNPs from SFTPC, rs4715 and rs1124; and 2 SNPs from SFTPD, rs721917 and rs2243639. The SNP ID, other used name, nucleotide change, and association with human disease as well as related references are given in Table 1. The genotype frequencies of these SNPs in mild and moderate CF compared to controls are given in Supplementary Table 2.

**Table 1:** Genetic variations of surfactant proteins SFTPA1, SFTPA2, SFTPB, SFTPC, and SFTPD association with disease.

**Gene**	**Variation ID**	**Other used name**	**Nucleotide**	**Disease association**	**References**
SFTPA2^*^	rs1059046	aa9 Asn/Thr	A/C	Respiratory syncytial virus (RSV), Influenza, Asthma, Pneumonia infection	(46–50)
	rs17886395	aa91 Pro/Ala	C/G	RSV, Tuberculosis (TB), Asthma	(46, 48, 51, 52)
	rs1965707	aa140 Ser/Ser	C/T	Asthma	(46)
	rs1965708	aa223 Gln/Lys	C/A	TB, Allergic rhinitis, High altitude pulmonary edema, RSV, recurrent Urinary tract infection (UTI), Meningococcal infection, Influenza	(46, 48, 49, 51–59)
SFTPA1^*^	rs1059047	aa19 Ala/Val	C/T	Idiopathic pulmonary fibrosis (IPF), Pneumonia infection, Asthma, RSV	(46, 48, 51, 53, 56, 60)
	rs1136450	aa50 Leu/Val	C/G	IPF, Pneumonia infection, Asthma, TB	(46, 48, 61, 62)
	rs1136451	aa62 Pro/Pro	G/A	TB, IPF, Chronic obstructive pulmonary disease (COPD)	(17, 52, 61, 63)
	rs1059057	aa133 Thr/Thr	G/A	Asthma	(46)
	rs4253527	aa219 Arg/Trp	C/T	TB, IPF, Influenza, Asthma, Pneumonia infection	(46, 48, 52, 61–64)
SFTPB^*^	rs2077079^**^	CA-18, CA1022	C/A	RSV, Respiratory distress syndrome (RDS), COPD, Asthma	(65–70)
	rs3024798^**^	CA1013, CA2052	A/C	Invasive pneumococcal disease (IPD), RDS	(69, 71)
	rs1130866^†^	TC1580, TC2619 aa131 Ile/Thr	T/C	COPD, Influenza, Pneumonia, RSV, Systemic Sclerosis, Acute lung injury	(50, 64, 65, 67–70, 72–84)
	rs7316^**^	AG9306, AG10345	A/G	Acute lung injury, RDS	(77, 85)
SFTPC^‡^	rs4715	aa138 Asn/Thr	A/C	RDS, Perinatal respiratory disease (PRD)	(86–89)
	rs1124	aa186 Asn/Ser	A/G	RDS, RSV, Asthma	(86, 88–90)
SFTPD^‡^	rs721917	aa11 Met/Thr	T/C	TB, Allergic rhinitis (AR), Asthma, COPD, RSV, Coronary artery stenosis, Bronchopulmonary dysplasia (BPD), RDS, Axial spondyloarthritis, Atherosclerosis, Sjogren syndrome, Interstitial lung diseases (ILDs), Type 2 diabetes (T2D), Inflammatory bowel disease (IBD)	(91–123)
	rs2243639	aa160 Thr/Ala	A/G	RSV, Ulcerative colitis (UC), BPD, RDS, COPD, IBD	(16, 54, 73, 77, 93, 94, 99, 101, 102, 106, 109, 111, 124–132)

* *Numbering of amino acids in SFTPA2, SFTPA1, and SFTPB, is that of the precursor molecule i.e. includes the signal peptide.Numbering of amino acids in SFTPC and SFTPD is based on the mature protein and does not include the signal peptide*.

** *All the SNP changes are located within the exons, except the three SFTPB marked with. The SFTPB a) rs2077079 is located 10 nt downstream of TATAA box, 5′ regulatory region (133); b) rs3024798 is located in the intron at the splice sequence of intron 2-exon 3 (65); and c) rs7316 is located in the 3′UTR 4 nucleotides upstream of the TAATAA polyadenylation signal (133). The SFTPB rs1130866 marked with is located within a potential N-linked glycosylation site, which has been shown to be glycosylated (134)*.

### Genotype analysis

The PCR-based RFLP or cRFLP (135) method was used for genotyping as described in previous publications for SFTPA1, SFTPA2, and SFTPD (136, 137), SFTPB (133, 137), and SFTPC (61). The PCR primer sequences and restriction enzymes used are given in Table 2. Briefly, PCRs were performed at 95°C for 2 min, 5 cycles of 95°C for 30 seconds, 50°C for 1 min, and 70°C for 1 min, then 25 cycles of 95°C for 30 s, 55°C for 1 min, and 70°C for 1 min, followed by an extension at 70°C for 4 min. Five microliter of each PCR products were used for appropriate restriction enzyme digestion (Table 2). The digested PCR product was separated by 8 or 10% of PAGE gel (based on the length of digested PCR fragments). The genotyping was done blindly. As samples (CF and Controls) were received, each was given a sequential laboratory number with no other identifiers and were genotyped all together without knowledge as to which sample is CF or Control. Therefore we believe that there was no bias in the genotyping. The genotype was scored based on the pattern of the digested PCR products for each sample.

**Table 2:** PCR primers for the SNPs Study.

**Gene**	**Variation ID**	**Primer sequence 5**^**′**^**- 3**^**′**^		**Restriction enzyme**	**References**
SFTPA2	rs1059046	F	GCT GTG CCC TCT GGC CCT Ta	Tru 91	(136)
		R	TCC TTT GAC ACC ATC TC		
	rs17886395	F	AGA GCG TGG AGA GAA GGG GcA	Bbv I	
		R	GGG TTT GTC TGA TCC CCA TC		
	rs1965707	F	CAT AAT GAC AGT AGG AGA GAA GGT CTT CTC	Bfa I	
		R	ACC CTC AGT CAG GCC TAC AT		
	rs1965708	F	GGA GCC TGC AGG TCG GGG AAA Atc G	αTaq I	
		R	TCA GAA CTC ACA GAT GGT CA		
SFTPA1	rs1059047	F	ACC TCA TCT TGA TGT CAG CCT CTG GTG CaG	Bbv I	(136)
		R	AGG GCC CAG GTC TCC TCT GA		
	rs1136450	F	ACC TCA TCT TGA TGT CAG CCT CTG GTG CaG	Dde I	
		R	AGG GCC AGG GTC TCC TcT GA		
	rs1136451	F	TTT TCT CTG CAG GCC CCA TGG GTg C	Hha I	
		R	GGG TTT GTC TGA TCC CCA TC		
	rs1059057	F	TCT GCA GGG CTC CAT ATT Gc	Msp I	
		R	CAC ACA CTG CTC TTT TCC tC		
	rs4253527	F	TCT GCA GGG CTC CAT ATT Gc	αTaq I	
		R	CAC ACA CTG CTC TTT TCC tC		(133)
SFTPB	rs2077079	F	GTC CAG CTA TAA GGG GCC GTG	ApaL I	
		R	GTG AGT GGT GGA GCT GCC TA		
	rs3024798	F	ACT CTT GTG TCC TCC ACC TTG	Nla III	
		R	GGC ATA GGT CAT CCT GGG CA		
	rs1130866	F	CTC GAA TTC ACT CGT GAA CTC CAG CAC CC	Dde I	
		R	GTG AGC TTG CAG CCC TCT CA		
	rs7316	F	CTG TGT AAT ACA ATG TCT GCA CTA	Bfa I	
		R	CTC GAA TTC TGC TGG GAT TGC AGG TGT GA		
SFTPC	rs4715	F	GCT GAT CGC CTA CAA GCC CAG	Spe I	(61)
		R	CTG GAA GTT GTG GAC TTT aCT A		
	rs1124	F	GAT GGA ATG CTC TCT GCA GG	Sac I	
		R	GCA CCT CGC CAC ACA GGG aG		
SFTPD	rs721917	F	CTC CTC TCT GCA CTG GTC AT	Fsp I	(136)
		R	ACC AGG GTG CAA GCA CTG cG		
	rs2243639	F	AGC GTG GAG TCC CTG GAA gC	Hha I	
		R	AGA TTC TCT CCA TGT TCC CAG		

*Low case letters: mismatch to DNA sequence*.

### Statistical analysis

We used the Wang et al.'s (138) approach (provides computer code (written in R) for public use), which is a more efficient approach compared to more traditional methods (139) to test and estimate genetic effects of each pair of the 17 SNPs. This approach integrates the principle of quantitative genetics, enabling the decomposition of the overall genetic effect into different components: the additive (a) and dominant genetic effects (d) of each SNP and additive x additive (aa), additive x dominant (ad), dominant x additive (da), and dominant x dominant epistatic effects (dd) between the two SNPs. By estimating the role of each of these components, this approach can provide a better understanding of inheritance mode by which SNPs impact the disease. By analyzing each SNP pair, we calculated *p*-values for each genetic component. A Bonferroni correction was used to adjust for multiple comparisons.

## Results

### Association of the surfactant protein genes with CF

Associations of single SNPs or SNP-SNP interactions with CF discussed below are shown in Tables 3, 4. The SNPs studied here are not rare alleles (136). The column “interaction type,” in Table 3 as well as in subsequent relevant tables, is the type of the SNP-SNP interaction (interaction between two SNPs within a given gene - intragenic, or interaction between SNPs of two genes-intergenic). The letter a or d is for additive effect or dominant effect. The number 1 or 2 is for the SNP1 or SNP2. In Table 3 the d1 stands for a dominant effect for SNP1, and d2 stands for a dominant effect for SNP2. If it is a1d2 (Table 4), the interaction type is additive effect for SNP1 and dominant effect for SNP2. For example in Table 4, (1) SFTPC rs1124 has a significant dominant effect (d2) (*P* = 0.0053). This means that the heterozygote is beyond the mean of two homozygotes in the degree of severity at this SNP. (2) SFTPA1 rs1059047 × SFTPC rs1124 has a significant additive x dominant epistatic effect (a1d2) (*P* = 0.0014). This means that the combination of the homozygote at the first SNP and the heterozygote at the second SNP is significantly different from other combinations. (3) SFTPA1 rs1059047 × SFTPC rs1124 has a significant dominant x dominant epistatic effect (d1d2) (*P* = 0.0021). This means that the combination of the heterozygote at the first SNP and the heterozygote at the second SNP is significantly different from other combinations. In general, it looks like SFTPB and SFTPC have a dominant effect in most of the interactions, whereas the genes encoding the hydrophilic proteins exhibit primarily an additive effect (Table 4). We speculate that the surfactant related functions imparted by SP-B and SP-C variants play a critical differential role in pulmonary CF and that functions imparted by SP-A1/SP-A2 and SP-D variants further enhance the CF disease progression.

**Table 3:** Genetic association of surfactant protein genes SFTPA1, SFTPB, and SFTPC with CF.

	**Gene**	**SNP #1 ID**	**SNP #2 ID**	**Interaction type**	***x*^2^**	***p*-value**
**Genetic association of STFPB and SFTPC with CF by a single SNP**						
1	SFTPB	rs7316		d	6.9689	0.0083
2	SFTPC	rs1124		d	5.8674	0.0154
**Genetic association of SFTPB and SFTPA1 with CF by intragenic SNP interaction**						
3	SFTPB	rs2077079	rs3024798	d1	3.2688	0.0325
4	SFTPA1	rs1136451	rs1059057	d2	2.7329	0.0469
5	SFTPA1	rs1136451	rs4253527	d2	3.5625	0.0238

*Interaction type: d - dominant, in single SNPs*.

*Number 1 and 2 in interaction type column; represent SNP1 and SNP2*.

*The d1 stands for a dominant effect for SNP1, and d2 stands for a dominant effect for SNP2*.

**Table 4:** Genetic association of surfactant protein genes SFTPA1, SFTPA2, SFTPB, SFTPC, and SFTPD with CF by intergenic interactions.

**Interaction #**	**SNP #1**		**SNP #2**		**Interaction type**	***x*^2^**	***p*-value**
	**Gene name**	**SNP ID**	**Gene name**	**SNP ID**			
1	SFTPA1	rs1136450	SFTPA2	rs1059046	a1d2	3.1699	0.0485
2	SFTPA1	rs4253527	SFTPA2	rs1059046	a1	5.1800	0.0233
3	SFTPA1	rs1136450	SFTPB	rs7316	d1d2	2.7135	0.0213
4	SFTPA1	rs1059057	SFTPB	rs7316	d2	3.1499	0.0332
5	SFTPA1	rs4253527	SFTPB	rs7316	d2	3.2619	0.0303
6	SFTPA1	rs1059047	SFTPC	rs1124	d2	5.6874	0.0053
					a1d2	7.8947	0.0014
					d1d2	4.6019	0.0021
7	SFTPA1	rs1136451	SFTPC	rs1124	d2	6.1400	0.0007
					a1d2	6.5633	0.0449
					d1d2	3.4875	0.0027
8	SFTPA1	rs1059057	SFTPC	rs1124	d2	6.1715	0.0007
					d1	4.4217	0.0104
					a1d2	8.4508	0.0146
					d1d2	5.3013	0.0293
9	SFTPA1	rs1059047	SFTPC	rs4715	a1d2	3.1655	0.0487
10	SFTPA1	rs1136450	SFTPC	rs4715	a1d2	3.5583	0.0182
11	SFTPA1	rs1136450	SFTPC	rs1124	a1d2	4.1079	0.0187
12	SFTPA1	rs1136451	SFTPC	rs4715	d2	3.2576	0.0328
					a1d2	4.8166	0.0236
					d1d2	2.6958	0.0218
13	SFTPA1	rs4253527	SFTPC	rs1124	d2	2.2285	0.0404
14	SFTPA2	rs1059046	SFTPB	rs1130866	a1d2	3.4707	0.0390
15	SFTPA2	rs1965707	SFTPB	rs2077079	d1d2	4.1099	0.0043
16	SFTPA2	rs1965708	SFTPB	rs2077079	d1d2	2.4405	0.0262
17	SFTPA2	rs1965708	SFTPB	rs7316	d2	2.4172	0.0331
18	SFTPA2	rs17886395	SFTPC	rs1124	a1d2	6.4974	0.0038
					d2	3.6608	0.0257
19	SFTPA2	rs1059046	SFTPC	rs4715	a1d2	4.1992	0.0227
20	SFTPA2	rs1059046	SFTPC	rs1124	d2	2.9051	0.0471
					a1d2	4.1161	0.0221
21	SFTPB	rs2077079	SFTPC	rs1124	a1d2	3.5371	0.0344
22	SFTPB	rs1130866	SFTPC	rs4715	d1a2	4.6271	0.0121
23	SFTPB	rs1130866	SFTPC	rs1124	d2	3.9303	0.0207
24	SFTPB	rs7316	SFTPC	rs1124	d2	2.4947	0.0303
					a1d2	2.4947	0.0392
25	SFTPB	rs2077079	SFTPD	rs2243639	a1a2	8.3123	0.0058
26	SFTPB	rs3024798	SFTPD	rs2243639	a1a2	7.0808	0.0058
					a2	3.5108	0.0311
27	SFTPB	rs7316	SFTPD	rs2243639	d1d2	2.6436	0.0094
					d2	2.0495	0.0494
28	SFTPB	rs2077079	SFTPD	rs721917	a1a2	5.4000	0.0108
					d1d2	2.6197	0.0306
29	SFTPB	rs3024798	SFTPD	rs721917	a1a2	4.4454	0.0398
					d1d2	2.5095	0.0242
					d1a2	4.0265	0.0151
30	SFTPB	rs1130866	SFTPD	rs721917	d1a2	3.4263	0.0193
31	SFTPB	rs1130866	SFTPD	rs2243639	d2	3.1616	0.0382
					d1a2	3.0764	0.0347
32	SFTPC	rs4715	SFTPD	rs721917	d1a2	5.8333	0.0049
33	SFTPC	rs1124	SFTPD	rs721917	d1	5.0904	0.0075
					d1a2	4.5151	0.0099
34	SFTPC	rs1124	SFTPD	rs2243639	d1	4.4393	0.0247

*A total of 34 interactions of the 5 surfactant protein genes are associated with CF. Interaction type: a - additive, d - dominant, ad - additive x dominant, da - dominant x additive, and dd - dominant x dominant between the two SNPs*.

*Number 1 and 2 in interaction type column; represent SNP1 and SNP2*.

*The a1 stands for a additive effect for SNP1 and a2 stands for additive effect for SNP2*.

*The d1 stands for a dominant effect for SNP1, and d2 stands for a dominant effect for SNP2*.

*The a1d2 stands for the interaction type is additive effect for SNP1 and dominant effect for SNP2*.

*The d1a2 stands for the interaction type is dominant effect for SNP1 and additive effect for SNP2*.

#### Single SNP or intragenic interactions

The data in Table 3 showed that (1) SFTPB SNP rs7316 is associated with CF (*X*^2^ = 6.9689, *p* = 0.0083); (2) another two SFTPB SNPs rs2077079 and rs3024798 are associated with CF through an intragenic interaction (*X*^2^ = 3.2688, *p* = 0.0325). (3) SFTPC SNP rs1124 is associated with CF (*X*^2^ = 5.8674, *p* = 0.0154); (4). Although no single SFTPA1 SNP by itself is associated with CF, the SFTPA1 rs1136451 SNP in an intragenic interaction with either SNP rs1059057 or rs4253527 is shown to associate with CF (*X*^2^ = 2.7329, 9 = 0.0469 or *X*^2^ = 3.5625, *p* = 0.0238, respectively) (Table 3).

#### Intergenic interactions among the surfactant protein genes

A total of 34 intergenic interactions of different combinations between SNPs of the studied genes were observed to associate with CF after Bonferroni correction. Significant interactions that include each of the studied genes are as follows: 19 interactions for SFTPB (X^2^ is 2.045–8.3123, *p* = 0.0398–0.0043); 18 interactions for SFTPC (X^2^ is *2.2285–8.4508, p* = 0.0487–0.0007); 13 interactions for SFTPA1 (X^2^ is 2.2285–7.8947, *p* = 0.0487–0.0007), 9 interactions for SFTPA2 (X^2^ is 2.4172–6.4974, *p* = 0.0485–0.0038), and 10 interactions for SFTPD (X^2^ is 2.2285–8.4508, *p* = 0.0487–0.0007). Below, we present significant interactions where each interaction contains a SNP from a given gene.

##### Intergenic interactions that contain SFTPB SNPs

**Figure 1:**
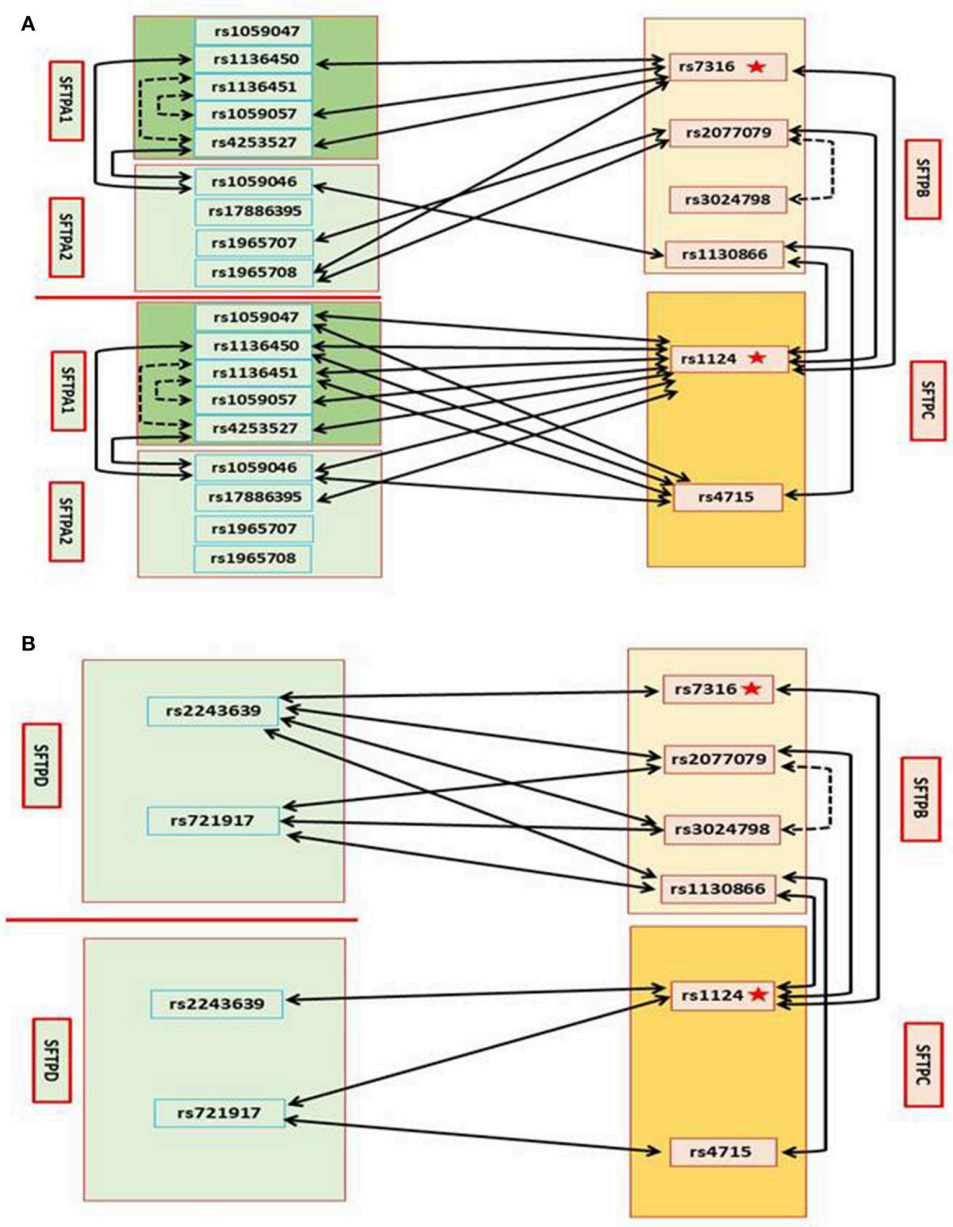
Genetic interactions between SP genes and associations with CF **(A)**. Genetic association of SFTPB and SFTPC with CF by single SNP, and intragenic and intergenic SNP-SNP interactions between SFTPB and SFTPC, and with SFTPA1 and SFTPA2. On the left SNPs of the surfactant genes SFTPA1 and SFTPA2 encoding the hydrophilic surfactant proteins, SP-A1 and SP-A2 and on the right SNPs of the surfactant genes SFTPB and SFTPC, encoding the hydrophobic proteins, SP-B and SP-C, are shown **(B)**. Genetic association of SFTPB and SFTPC with CF by single SNP, and intragenic and intergenic SNP-SNP interactions between SFTPB and SFTPC, and with SFTPD. On the left SNPs of SFTPD and on the right SNPs of SFTPB and SFTPC are shown: In Figure, the star depicts a single SNP association with CF, the black dash line depicts intragenic interactions associated with CF, and the black solid line depicts intergenic interactions associated with CF.

All of the 4 studied SFTPB SNPs, rs7316, rs2077679, rs3024798, and rs1130866, are associated with CF through 18 intergenic SNP-SNP interactions with 3 SNPs in SFTPA1, 4 SNPs in SFTPA2, 4 SNPs in SFTPC, and 7 SNPs in SFTPD **(**X^2^ is 2.045–8.3123, *p* = 0.0398–0.0043) (Table 4). Of the 18 intergenic SNP-SNP interactions that included SFTPB SNPs, only 4 contain SNPs from SFTPC encoding the other hydrophobic surfactant protein, whereas 14 interactions are with genes encoding the hydrophilic surfactant proteins, seven are with SFTPA (3 for SFTPA1 and 4 for SFTPA2) (Figure 1A) and seven with SFTPD (Figure 1B).

SFTPB SNP rs7316 is associated with CF by itself as noted above (Table 3), and by intergenic interactions with 3 SNPs in SFTPA1 (*n* = 3), one SNP in SFTPA2, one SNP in SFTPC, and one SNP in SFTPD. SFTPB SNP rs2077079 is associated with CF by intragenic interaction with rs3024798, and by intergenic interaction with SNPs in SFTPA2 (*n* = 2), SFTPC (*n* = 1), and SFTPD (*n* = 2). SFTPB SNP rs3024798 is associated with CF, in addition to the intragenic interaction with rs2077079 (noted above), by intergenic interactions with SFTPD (*n* = 2). SFTPB SNP rs1130866 is associated with CF by intergenic interaction with SFTPA2 (*n* = 1; Figure 1A), SFTPC (*n* = 2), and SFTPD (*n* = 2; Figure 1B).

##### Intergenic interactions that contain SFTPC SNPs

Both of the SFTPC SNPs studied, rs4715 and rs1124, are associated with CF through 18 intergenic SNP-SNP interactions with SNPs in SFTPA1, SFTPA2, SFTPC, and SFTPD (X^2^ is 2.2285–8.4508, *p* = 0.0487–0.0007) (Table 4). Of the 18 intergenic SNP-SNP interactions associated with CF, eight are with SFTPA1, three are with SFTPA2, four are with SFTPB (Figure 1A), and three are with SFTPD (Figure 1B).

Of the 18 intergenic SFTPC SNP-SNP interactions, only 4 are with SFTPB the gene that encodes the other hydrophobic surfactant protein, whereas 14 interactions are with genes encoding the hydrophilic surfactant proteins (8 for SFTPA1, 3 for SFTPA2, and 3 for SFTPD). SNP rs1124 as noted above (Table 3) is associated with CF by itself, and by 11 intergenic SNP-SNP interactions of the surfactant protein genes, SFTPA1, SFTPA2, SFTPB (Figure 1A), and SFTPD (Figure 1B). SNP rs4715 is associated with CF by 7 intergenic SNP-SNP interactions with SFTPA1, SFTPA2, SFTPB (Figure 1A), and SFTPD (Figure 1B).

##### Intergenic interactions that contain SFTPA1 SNPs

All of the 5 studied SFTPA1 SNPs are associated with CF through 13 intergenic SNP-SNP interactions with SNPs in SFTPA2 (*n* = 2), SFTPB (*n* = 3), and SFTPC (*n* = 8) (X^2^ is 2.2285–7.8947, *p* = 0.0487–0.0007) (Table 4, Figure 1A). Of interest, no SNP-SNP interaction was observed with SFTPD. Each SFTPA1 SNP is shown to have 1–3 intergenic SNP interactions with the other surfactant protein genes. 1) SNP rs1136451 exhibits a significant association with CF through interactions with another two SFTPA1 SNPs (rs1059057 and rs4253527), as well as with both SFTPC SNPs (rs1124 and rs4715). 2) SNPs rs1136450 and rs4253527 are associated with CF through interaction with SNP rs1059046 of the SFTPA2.

Of the 13 intergenic SFTPA1 SNP-SNP interactions, 11 are with SFTPB and SFTPC encoding the hydrophobic surfactant proteins, and only two interactions are with SFTPA2 that encodes the hydrophilic surfactant protein A2 (Figure 1A), whereas no interactions are observed with SFTPD.

##### Intergenic interactions that contain SFTPA2 SNPs

Each SFTPA2 SNP is shown to have 1–3 intergenic SNP interactions with other surfactant protein genes. Table 4 shows that the SFTPA2 gene is associated with CF through 9 intergenic SNP-SNP interactions with SFTPA1 (*n* = 2), SFTPB (*n* = 4), and SFTPC (*n* = 3) **(**X^2^ is 2.4172–6.4974, *p* = 0.0485–0.0038, Figure 1A). Similarly to SFTPA1, no interaction of SFTPA2 with SFTPD was found to be associated with CF. SFTPA2 SNP rs1059046 appears to stand out from the other SFTPA2 SNPs studied, as this SNP (1) is associated with CF via five of the total nine interactions observed with the other SP genes, (2) is the only SNP that shows interactions with two SFTPA1 SNPs, and (3) shows interactions with both hydrophobic surfactant proteins SFTPB and SFTPC (Figure 1A). Of the 9 intergenic interactions, 7 are with the SFTPB and SFTPC hydrophobic surfactant proteins genes, and only two interactions are with the hydrophilic surfactant protein gene, SFTPA1, and no interactions were observed with SFTPD.

##### Intergenic interactions that contain SFTPD SNPs

Table 4 shows that SFTPD is associated with CF through 10 intergenic SNP-SNP interactions with SNPs in SFTPB and SFTPC, but as noted above no interactions were observed with SFTPA1 or SFTPA2. Of the 10 intergenic SFTPD interactions associated with CF, seven of these are with SFTPB and three with SFTPC (X^2^ is 2.2285–8.4508, *p* = 0.0487–0.0007) (Figure 1B). SFTPD SNP rs721917 is associated with CF through 5 intergenic interactions with SFTPB (*n* = 3) and SFTPC (*n* = 2); rs2243639 is associated with CF also through 5 intergenic interactions but four of these are with SFTPB and only one with SFTPC (Figure 1B).

**Figure 2:**
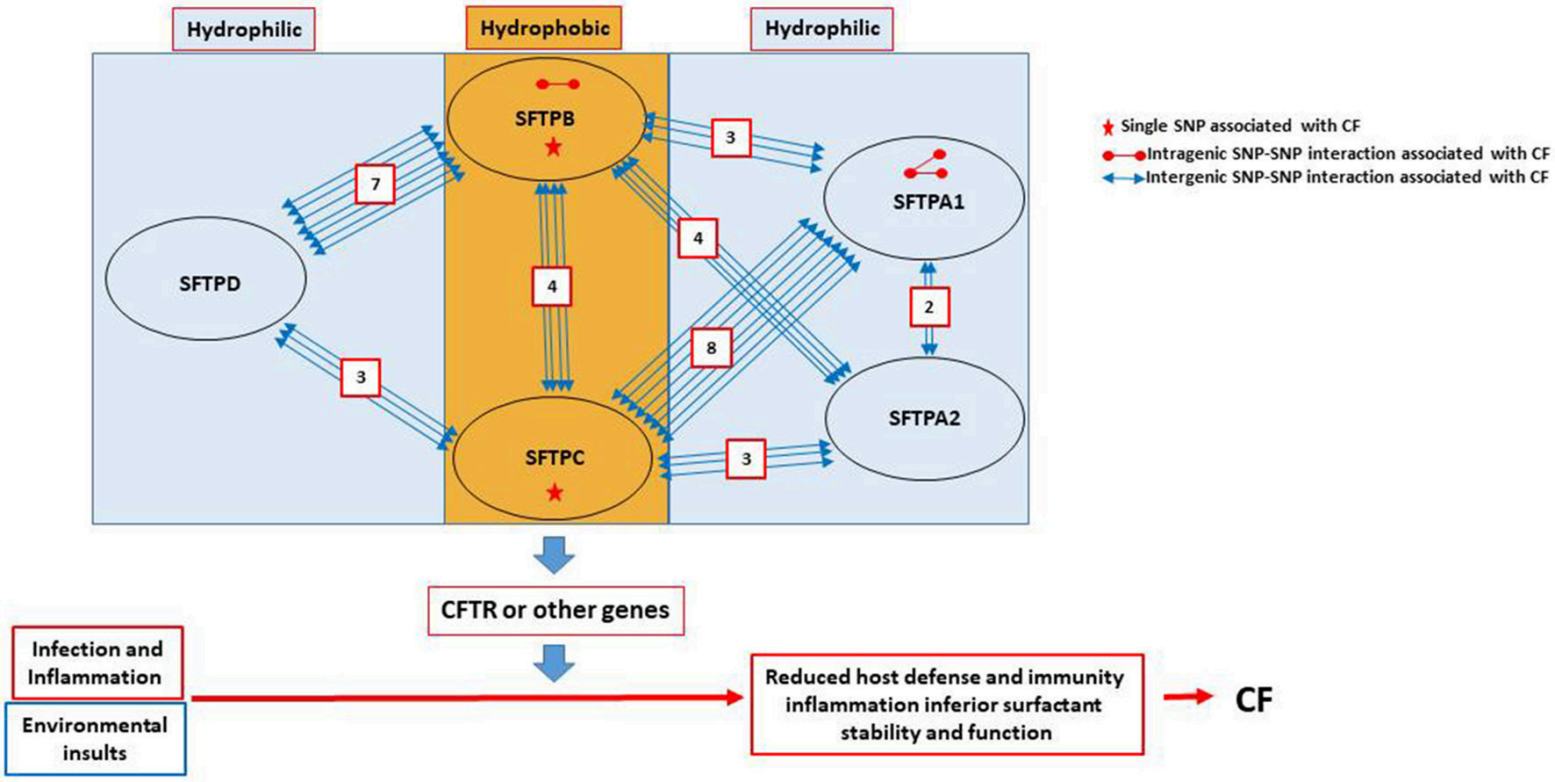
Summary of SNP interactions between genes encoding the surfactant proteins. The cartoon in this figure depicts the observed associations and reveals the following: (a) No association between SFTPD and SFTPA1 or SFTPA2. (b) Out of the 34 intergenic interactions, 28 are between genes encoding the hydrophobic and the hydrophilic surfactant proteins. Only four are between genes (SFTPB and SFTPC) encoding the hydrophobic surfactant proteins and two are between genes (SFTPA1 and SFTPA2) encoding the hydrophilic SP-A1 and SP-A2. (c) SFTPC has the highest number of interactions with SFTPA1 (*n* = 8), while SFTPB has the highest number of interactions with SFTPD (*n* = 7). (d) SFTPB has a total of 14 intergenic interactions with genes encoding hydrophilic proteins, seven with SFTPD, four with SFTPA2, and three with SFTPA1. SFTPC also has 14 intergenic interactions, eight with SFTPA1, three with SFTPA2 and only 3 with SFTPD. (e) SFTPA1 has two intergenic interactions with SFTPA2, as well as intragenic interactions. (f) SFTPB is the only other gene (other than SFTPA1) that has an intragenic interaction. (g) Only SFTPB and SFTPC have each a single SNP association with CF.

In summary, when we studied the entire CF cohort, we observed a) Two SNPs (one from SFTPB and the other from SFTPC) to be individually associated with CF; b) Three intragenic interactions (2 of SFTPA1 and one of SFTPB) to associate with CF; c) A total of 34 intergenic interactions of different combinations between the various genes studied, except between SFTPD and SFTPA1 or SFTPA2, to associate with CF; A summary of all the significant intragenic and intergenic interactions is shown in Supplementary Table 3, and Figure 2. Moreover, our results have a potential clinical impact. For example, since SFTPA1 rs1059047 x SFTPC rs1124 has a significant additive x dominant epistatic effect (a1d2) (*P* = 0.0014), the combination of the homozygote at the first SNP and the heterozygote at the second SNP is significantly different from other combinations. Thus, we can make a prediction of patients' severity based on their genotypes at these two SNPs.

### Association of the surfactant protein genes with CF disease severity subgroups

To gain insight into the contribution of the SP genes to CF disease severity, we separated the CF cohort into two subgroups, mild (*n* = 64) and moderate/severe (*n* = 15). The data showed that after Bonferroni correction a single SFTPB SNP (rs7316) to be associated with mild CF and no other SNPs were found to associate with either CF subgroup. However, there are a number of intergenic SNP interactions (*p* < 0.01 prior to Bonferroni correction) that associated with each CF subgroups (Supplementary Table 4).

**Figure 3:**
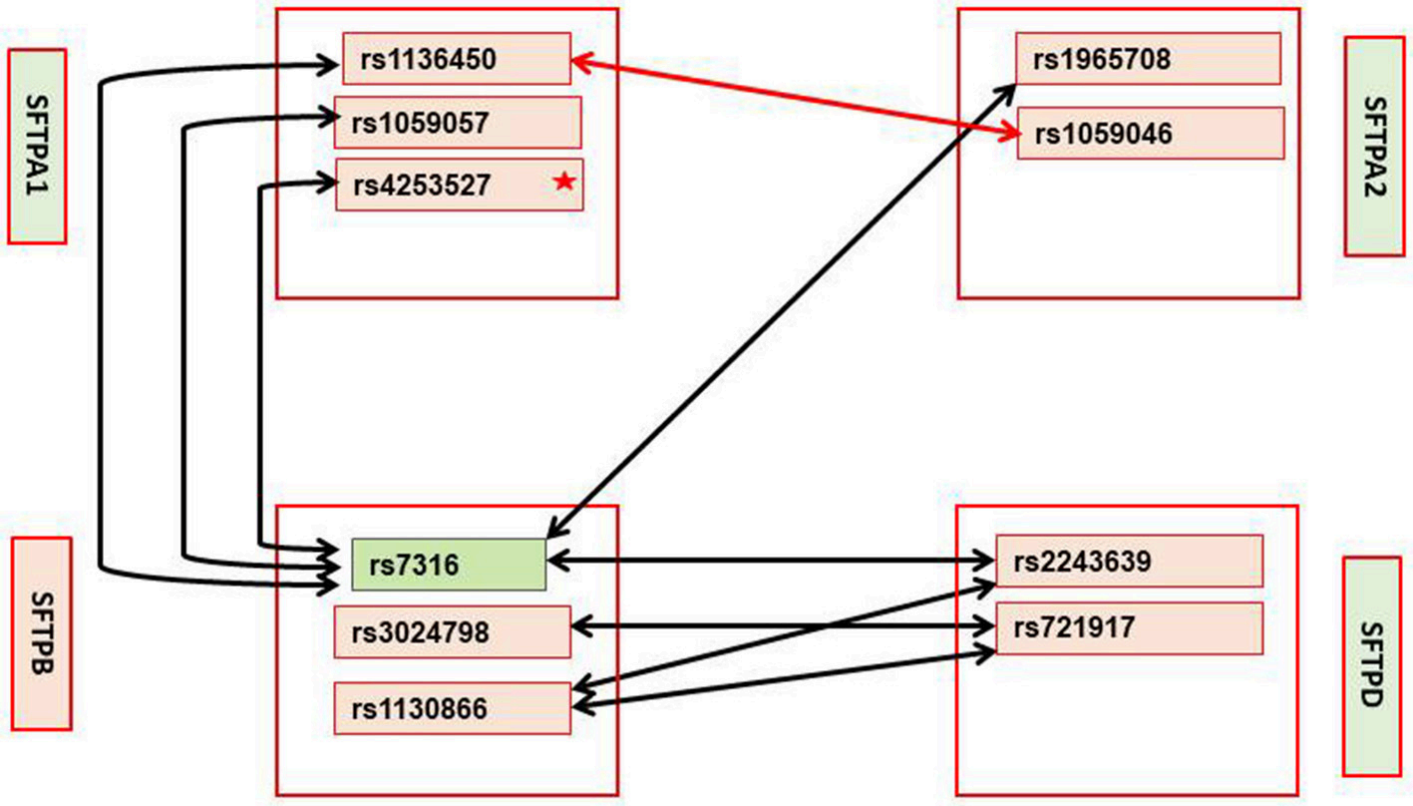
Interactions in mild and moderate/severe CF subgroups with p-value < 0.01; these interactions were significant in the entire CF group after Bonferroni correction. *Mild CF:* Significant interactions between SFTPA1, SFTPA2, SFTPB, and SFTPD are shown with black arrows. Green box indicates the only SNP that remained significant after Bonferroni correction. Star (*) indicates that this SNP is significant at *p* < 0.03 by itself. Moderate/severe CF: Red arrow shows the only significant interaction at *p* < 0.01 that is also significant in the entire CF groups after Bonferroni correction. No significant interactions that include SNPs of the SFTPC are observed in either severity group.

Given the smaller number of subjects in each CF subgroup, and as we wished to gain further insight into the interactions observed, we focused our attention on significant associations (*p* < 0.01) observed in each subgroup that were also significant after Bonferroni correction in the entire CF group. These are shown in Table 5 and Figure 3. Eight intergenic interactions were observed for the mild subgroup and only one for the moderate/severe subgroup. In the mild group, SNPs of the SFTPB exhibited the same number of interactions with SFTPD (*n* = 4) as they did with SFTPA1+SFTPA2 (*n* = 4). More interactions were observed between SFTPB and SFTPA1 (*n* = 3) than SFTPA2 (*n* = 1) in the mild subgroup. No significant associations with SFTPC were observed with either disease severity group. In addition similar to the entire CF group, no associations were found between SFTPD and SFTPA1 or SFTPA2 in either subgroup.

**Table 5:** Interactions in mild and moderate/severe CF subgroups with *p* < 0.01.

**Interaction #**	**SNP #1**		**SNP #2**		**Interaction type**	***x*^2^**	***p*-value**
1	SFTPB	rs7316	SFTPA2	rs1965708	d1	3.66565180	0.00820001
2	SFTPB	rs7316	SFTPA1	rs1136450	d1d2	3.81926684	0.00607927
3	SFTPB	rs7316	SFTPA1	rs1059057	d1	4.77551020	0.00931902
4	SFTPB	rs7316	SFTPA1	rs4253527	d1	5.55638114	0.00510446
5	SFTPB	rs3024798	SFTPD	rs721917	d1d2	5.47559633	0.00077503
6	SFTPB	rs1130866	SFTPD	rs721917	d1a2	6.90124062	0.00057516
7	SFTPB	rs1130866	SFTPD	rs2243639	d1a2	5.83841692	0.00299812
8	SFTPB	rs7316	SFTPD	rs2243639	d1d2	3.49498711	0.00210045
9^*^	SFTPA2	rs1059046	SFTPA1	rs1136450	a1	2.29978355	0.00205943

*These were significant after Bonferroni correction in the entire CF group*.

Interactions in mild subgroup (1–8);

* *Interaction in moderate/severe subgroup*.

*Interaction type: a-additive, d- dominant, da - dominant x additive, and dd - dominant x dominant between the two SNPs*.

*Number 1 and 2 in interaction type column; represent SNP #1 and SNP #2*.

*The a1 stands for a additive effect for SNP1 and a2 stands for additive effect for SNP2*.

*The d1 stands for a dominant effect for SNP1, and d2 stands for a dominant effect for SNP2*.

*The d1a2 stands for the interaction type is dominant effect for SNP1 and additive effect for SNP2*.

## Discussion

In this study, we investigated the genetic contribution of the surfactant protein genes, SFTPA1, SFTPA2, SFTPB, SFTPC, and SFTPD to CF and disease severity subgroups by genetic association analysis of single SNP and intragenic and intergenic SNP-SNP interactions. (a) For the entire CF group, we observed that all 5 surfactant protein genes are associated with CF by single SNP association, intragenic (Table 3), and/or intergenic SNP-SNP interactions (Table 4).( b) For the CF severity subgroups, we observed after Bonferroni correction a single SFTPB SNP to be associated with mild CF and several interactions (*p* < 0.01) to associate with mild or moderate/severe CF subgroups (Supplementary Table 4 and Table 5).

Human diseases are complex and determined by environmental factors and genes. Single genetic mutations or multiple mutations in a single gene, constitute a part or a small part of disease mechanisms. To study the full spectrum of gene(s) contributing to a disease either by being the primary cause or by modifying disease expression, an integrated genetic approach is needed to understand genetic control and clinical therapy. By integrating quantitative genetic principles (138) a statistical method was developed to test associations of pairwise SNPs and disease in a case-control setting. This method can decompose the overall genetic effect into its underlying components and test the significance of each component, gaining insight into the mechanisms of how SNPs affect disease. It has been used in our previous studies in human inflammatory bowel diseases (IBD) that included both case-control and case-trios studies, and targeting SNPs in one gene and in multiple genes of a metabolic pathway (140, 141). Because this method is powerful and helps understand genetic contribution to a disease via interactions of genes in a metabolic pathway or gene network, we used it in the present study.

### Association of the surfactant protein genes with the entire CF group

#### Association of the hydrophobic surfactant protein genes, SFTPB and SFTPC, with CF

Surfactant proteins SP-B and SP-C are hydrophobic membrane proteins that increase the rate that surfactant spreads over the alveolar surface, and are required for proper biophysical function of surfactant and lung function. SP-B is also important for SP-C processing, as indicated in SP-B deficient states where an aberrant SP-C was observed (7, 142, 143).

All four of the SFTPB SNPs studied showed significant interactions with SNPs of one or more SP genes. The rs7316 is located in the 3′-UTR and could affect regulation of polyadenylation (133) and has previously been associated with acute lung injury (77). This SFTPB SNP is not only significant by itself but interacts with SNPs of all 4 SP genes. The rs2077079 interacts with SNPs of all SP genes, except SFTPA1. It is located at 11 nt downstream of the TATA box and could affect gene transcription. The rs1130866, which also interacts with SNPs of SP genes, except SFTPA1, is a missense mutation that changes the encoded amino acid (Ile/Thr) and an N-linked glycosylation site of the protein, shown previously to be indeed glycosylated in the Thr-containing variant (1580_C) (134). This is a significant change and may be an important contributing factor in various diseases and/or in response to environmental oxidative stress. Moreover, the SFTPB 1580_C (rs1130866) genetic variant has been observed to be a risk factor in several lung disease, such as idiopathic pulmonary fibrosis (IPF) (61), chronic obstructive pulmonary disease (COPD) (17), acute respiratory distress syndrome (ARDS) (137), septic shock and those with risk of respiratory failure after community acquired pneumonia (144), as well as increases mortality, apoptosis, and lung injury in mice carrying the human SP-B 1580_C variant compared to those with the 1580-T variant (145). On the other hand, the SP-B1580-T/T (rs1130866) is associated with protection against interstitial lung disease (ILD) with systemic sclerosis (64). The rs3024798 is located within the splicing sequence of intron 2–exon 3, and although its effect on splicing is unknown (65) it has previously been associated with invasive pneumococcal disease (IPD) (71). This SNP, in addition to an intragenic interaction, is the only SNP that interacts with a single SP gene, the SFTPD. Together these SNP variations in SFTPB could affect SP-B function by altering N-linked glycosylation and/or affect regulation at several levels including transcription and splicing.

SP-C is a hydrophobic surfactant protein and plays an important role in surfactant function. Mutations in SP-C have also been shown to associate with a number of pulmonary diseases (15), such as ILD and pulmonary alveolar proteinosis (PAP). Both SFTPC variants are missense, where amino acids 186 and 138 are changed, Ser/Asn in rs1124, and Thr/Asn in rs4715, respectively. While both SNPs associate with CF through numerous intergenic interactions with the other SPs, the rs1124 is also associated with CF by itself. The potential mechanisms via which these may affect function are not known. The two SP-C variants (rs1124, Ser/Asn and rs4715, Thr/Asn) have previously been associated with RDS (86, 88, 89), and children infected with respiratory syncytial virus (RSV) (90).

In summary, the observations made indicate that the hydrophobic proteins, shown previously to be key in surfactant function and homeostasis and consequently in lung function may play a central role in CF. The only two individual SNPs to associate with CF by themselves were SNPs of SFTPB rs7316 (*p* = 0.0083) and SFTPC rs1124 (*p* = 0.0154) (indicated by star in Figure 1A). Moreover, of the 37 interactions shown to associate with CF, SFTPB or SFTPC was part of the 35 interactions. Given the importance of SP-B and SP-C in surfactant function and lung function, and the fact that lung function deterioration is a major issue in CF, it is likely that the SFTPB and SFTPC genes are modifier genes for CF lung function, by modulating surfactant structural organization and stability.

#### Association of hydrophilic surfactant protein genes SFTPA1/A2 and SFTPD with CF

The SP-A1, SP-A2, and SP-D mediate innate immunity in the lung and via interactions with the alveolar macrophage the sentinel cell of innate lung host defense, promote, among others, bacterial and viral phagocytosis, and cytokine production, as well as affect lung inflammatory processes. The mechanisms implicated in these processes may differ among these molecules (19). Of interest in the present study, there were no significant interactions between SNPs of SFTPD and SFTPA1 or SFTPA2 and only two significant intergenic interactions were observed between SFTPA1 and SFTPA2 (Figure 1A). A single SNP in SFTPA2 (rs1059046) interacted with two different SNPs in SFTPA1 (rs1136450, aa 50 Leu/Val and rs4253527, aa 219 Arg/Trp). The SFTPA2 SNP (rs1059046) changes the amino acid (Thr/Asn) at codon 9 of the precursor molecule which is part of the signal peptide, having the potential to affect processing of the SP-A2. This SNP was previously shown to associate with increased risk in RSV (47). The two SFTPA1 SNPs (rs1136450, rs4253527) that interact with this SFTPA2 SNP are located in the collagen-like domain and in the carbohydrate recognition domain (CRD), respectively, of the SP-A2 and change the encoded amino acid (rs1136450, aa 50 Leu/Val, rs4253527, aa 219 Arg/Trp). Moreover, higher- or lower-order of oligomerization of SP-A and SP-D is known to affect their functional capabilities (146–149), and has been observed that naturally occurring SP-A and SP-D oligomers have functional relevance in patients with chronic lung diseases such as CF (32, 35). Thus, each of the surfactant protein variants may differentially affect innate immune functions and in CF may each differentially modify lung host defense.

Multiple interactions between the hydrophobic and the hydrophilic protein genes were observed indicating that these two groups of proteins may co-operatively or synergistically contribute to the expression of pulmonary CF. As depicted in Figure 2, SFTPD SNPs are primarily found in interaction with SNPs of SFTPB (*n* = 7), whereas SNPs of SFTPA1 are primarily found in interactions with SNPs of SFTPC (*n* = 8). The latter is of interest because SP-A1 has been shown to affect more efficiently (compared to SP-A2) the structural organization of surfactant (10), indicating that SP-A1 and SP-C may cooperatively affect surfactant structure, which in turn may affect surfactant function and lung function. Moreover, the large number of SNP interactions between SFTPD and SFTPB is puzzling and not intuitively understood as far as surfactant structure or function is concerned. This is because, although SP-D is generally found in the bronchoalveolar lavage fluid and significantly less in alveolar epithelia, and grouped with SP-A1/A2 based on its structural similarity and function with these proteins (5, 6), it is not found in the surfactant lipoprotein complex.

### II. association of the surfactant protein genes with CF subgroups

When we studied disease severity subgroups (mild and moderate/severe), we found after Bonferroni correction a single SFTPB SNP (rs7316) to associate with mild CF. This SNP has also been shown to associate with acute lung injury (77). Lack of SP-B is not compatible with life, and SP-B deficiency affects SP-C processing (7, 142, 143). This SFTPB SNP is located within the 3′UTR and may affect regulation of polyadenylation (133). Thus, this SFTPB SNP may contribute to CF either by affecting its regulation (133) and/or by affecting SP-C processing (7, 142, 143). In the CF subgroups of the eight significant intergenic interactions (p < 0.01) that were also significant in the entire CF group after Bonferroni correction, seven were for the mild group and one for the moderate/severe subgroup. The ones for the mild group all were between SFTPB SNPs and SNPs of the genes encoding the hydrophilic surfactant proteins.

The SFTPB SNP (rs7316) that is significant by itself in mild CF after Bonferroni correction was the only SFTPB SNP that interacted with SNPs of SFTPA1 or SFTPA2, whereas the SNP-SNP intergenic interactions between SFTPB and SFTPD include three of the four SFTPB SNPs studied. These indicate the importance of SFTPB in mild CF which may provide protection in the sense of enabling a milder form of pulmonary CF through its surfactant-associated function or perhaps other currently unknown function. Based on the differences of SNP-SNP interactions; it is likely that the mechanisms via which the hydrophilic proteins contribute to mild CF may differ. The SFTPA1 SNP (rs4253527) was also significant by itself (*p* < 0.03) (Supplementary Table 4) in the mild CF group. SP-A and SP-D bind via their carbohydrate recognition domains (CRD) to the carbohydrate motifs on bacteria, viruses, fungi, etc. (150–152). Thus, SNPs in CRD may differentially affect binding to various ligands to trigger the host's innate immune response. The SFTPA1 SNP (rs4253527) is located in the CRD and changes the encoded amino acid (Arg/Trp) holding the potential to differentially affect innate immunity under various conditions including oxidative stress, since Trp is more sensitive to oxidation than Arg (33). In fact SP-A1 variants that differ in CRD at rs4253527 have been shown to differ in their ability to enhance phagocytosis (153) and cytokine production (154). Moreover, SP-A1 has been shown to more efficiently affect surfactant reorganization (than SP-A2) in the alveolar space (10). Whether this SNP provides protection in CF via its role in surfactant function or innate immunity or both remains to be determined. Recently, SP-A1 and SP-A2 have been shown to differentially affect lung mechanics (12) indicating another role of the SP-As beyond innate immunity.

For the moderate/severe group the only significant interaction is between the SNP rs1136450, (aa 50 Leu/Val) of SFTPA1 and SNP rs1059046 (aa 9 Thr/Asn) of SFTPA2. This interaction may be unique to moderate/severe disease group because it was not identified in the mild group even in interactions with *p* < 0.05 (Supplementary Table 4). Similarly, interactions 1, 3, 4, 7, and 8 in the mild group (Table 5) were not identified in the moderate/severe disease group even in interactions with *p* < 0.05 (Supplementary Table 4), indicating that these may be unique to the mild CF group.

In summary, a single SNP of the SFTPB is a marker for mild pulmonary disease in CF. A number of intergenic interactions that all include SNPs of SFTPB as well as a single intragenic SFTPA1 and SFTPA2 interaction are likely to be markers for mild and moderate/severe pulmonary disease in CF, respectively.

Limitations of the study include: (a) The small number of subjects especially after the CF group was divided in the mild and moderate/severe subgroups. This also precluded analysis of the individual components of FEV_1_ and FVC; (b) The limited clinical information; the samples were collected at an earlier time using only FEV_1_ as a discriminator for the severity subgroups. However, this is still the main biomarker to assess disease severity. (c) The lack of associations with bacterial strain and correction for age. (d) The CFTR mutation is not known although most of the subjects are expected to carry the ΔF508, which is approximately found in 70% of the CF patients.

However, in spite of these limitations the present findings indicate that both groups of surfactant proteins, those involved in innate immunity, and those affecting surfactant functions, associate with CF via complex interactions. These may contribute to pulmonary disease progression in CF, by affecting surfactant structure and function and/or by affecting innate immunity functions. Altered surfactant leads to a compromised lung function, and lung function deterioration is a major setback in CF pulmonary disease. Similarly host defense and inflammatory processes are partially affected in CF and SPs may play a role in these. Thus, based on the collective information with regards to their function and the observations made here, the SP genes are likely to be significant gene modifiers of CF and must be studied further. As gene modifiers, SPs may explain the varied progression of the pulmonary disease in CF in terms of lung function and host defense.

## Data availability

All the data are presented as supplementary files.

## Ethics statement

All protocols used in this study were evaluated and approved by the institutional review board from the Human Subject Protection Office of the Pennsylvania State University College of Medicine.

## Author contributions

ZL analyzed and synthesized the data, contributed to the manuscript writing. NT analyzed and synthesized data for CF subgroups and contributed to manuscript writing. RW performed statistical analysis and contributed to manuscript writing. SD performed all the genotyping. MY assisted with statistical analysis. NJT attended to human subjects issues, sample collection and contributed to manuscript writing. XL checked genotype data sheets after multiple transfers. TL reviewed literature and made Table 1. SW helped with clinical assessment. JF designed the study and provided oversight to the entire project, involved in data analysis, integration, and writing of the manuscript.

### Conflict of interest statement

The authors declare that the research was conducted in the absence of any commercial or financial relationships that could be construed as a potential conflict of interest. The reviewer AT and handling Editor declared their shared affiliation.

## Abbreviations

CF: Cystic fibrosis
CFTR: cystic fibrosis transmembrane conductance regulator
FEV_1_: Forced expired volume in 1 sec
SP-A: surfactant protein A
SFTPA1: gene encoding SP-A1
SFTPA2: gene encoding SP-A2
SFTPB: gene encoding SP-B
SFTPC: gene encoding SP-C
SFTPD: gene encoding SP-D
SNPs: single-nucleotide polymorphism.
