# Supplementary material for: Genetic Association of Pulmonary Surfactant Protein Genes, SFTPA1, SFTPA2, SFTPB, SFTPC, and SFTPD With Cystic Fibrosis
Source: Front Immunol. 2018 Oct 2;9:2256. doi: 10.3389/fimmu.2018.02256 (PMC6175982; doi:10.3389/fimmu.2018.02256)
Supplement: Supplementary file 1 [file Table_1.docx]

SUPPLEMENTARY TABLES

Supplementary Table 1. Study case-trio samples.

| **Family #** | **Sample #** | **Case** | **Mother** | **Father** | **Study Category** | **Gender** | **Race** | **Ethnic Group** | **Age Category** |
| --- | --- | --- | --- | --- | --- | --- | --- | --- | --- |
| 1 | 11244 | y |  |  | CF:Mild, Pseudomonas | Male | White | American | Adult |
|  | 11245 |  | y |  | CF:Parental Control | Female | White | American | Adult |
| 2 | 11294 | y |  |  | CF:Mild, Pseudomonas | Female | White | American | Child |
|  | 11295 |  | y |  | CF:Parental Control | Female | White | American | Adult |
|  | 11296 |  |  | y | CF:Parental Control | Male | White | American | Adult |
| 3 | 11213 | y |  |  | CF:Mild, Staph | Male | Hispanic | Mexican | Child |
|  | 11214 | y |  |  | CF:Mild, Pseudomonas | Male | Hispanic | Mexican | Child |
|  | 11215 |  | y |  | CF:Parental Control | Female | Hispanic | Mexican | Adult |
| 4 | 11234 | y |  |  | CF:Mild, Pseudomonas | Male | White | American | Child |
|  | 11235 |  | Y |  | CF:Parental Control | Female | White | American | Adult |
|  | 11236 |  |  | y | CF:Parental Control | Male | White | American | Adult |
| 5 | 11174 | y |  |  | CF:Mild, Staph | Male | White | American | Child |
|  | 11175 |  | y |  | CF:Parental Control | Female | White | American | Adult |
| 6 | 11184 | y |  |  | CF:Mild, Pseudomonas | Male | White | American | Child |
|  | 11185 |  | y |  | CF:Parental Control | Female | White | American | Adult |
|  | 11186 |  |  | y | CF:Parental Control | Male | White | American | Adult |
| 7 | 11144 | y |  |  | CF:Mild, Pseudomonas | Female | White | American | Child |
|  | 11145 |  | y |  | CF:Parental Control | Female | White | American | Adult |
|  | 11146 |  |  | y | CF:Parental Control | Male | White | American | Adult |
| 8 | 11013 | y |  |  | CF:Mild, Pseudomonas | Male | White | American | Child |
|  | 11015 |  | y |  | CF:Parental Control | Female | White | American | Adult |
| 9 | 11114 | y |  |  | CF:Mild, Pseudomonas | Female | White | American | Child |
|  | 11115 |  | y |  | CF:Parental Control | Female | White | American | Adult |
|  | 11116 |  |  | y | CF:Parental Control | Male | White | American | Adult |
| 10 | 11124 | y |  |  | CF:Moderate, Other | Male | White | American | Child |
|  | 11125 |  | y |  | CF:Parental Control | Female | White | American | Adult |
| 11 | 11134 | y |  |  | CF:Severe, Pseudomonas | Male | Hispanic | Mexican | Child |
|  | 11135 |  | y |  | CF:Parental Control | Female | Hispanic | Mexican | Adult |
| 12 | 11063 | Y |  |  | CF:Mild, Staph | Male | White | American | Child |
|  | 11064 | y |  |  | CF:Mild, Staph | Male | White | American | Child |
|  | 11066 |  |  | y | CF:Parental Control | Male | White | American | Adult |
|  | 11065 |  | y |  | CF:Parental Control | Female | White | American | Adult |
|  | 11067 |  | y |  | CF:Grandparent Control | Female | White | American | Adult |
| 13 | 11274 | y |  |  | CF:Mild, Staph | Male | White | American | Child |
|  | 11276 |  |  | y | CF:Parental Control | Male | White | American | Adult |
| 14 | 11034 | y |  |  | CF:Mild, Pseudomonas | Male | White | American | Child |
|  | 11035 |  | y |  | CF:Parental Control | Female | White | American | Adult |
| 15 | 11044 | y |  |  | CF:Mild, Pseudomonas | Male | White | American | Child |
|  | 11045 |  | y |  | CF:Parental Control | Female | White | American | Adult |
| 16 | 11384 | y |  |  | CF:Mild, Staph | Male | White | American | Child |
|  | 11385 |  | y |  | CF:Parental Control | Female | White | American | Adult |
|  | 11386 |  |  | y | CF:Parental Control | Male | White | American | Adult |
| 17 | 11444 | y |  |  | CF:Mild, Staph | Male | White | American | Child |
|  | 11445 |  | y |  | CF:Parental Control | Female | White | American | Adult |
|  | 11446 |  |  | y | CF:Parental Control | Male | White | American | Adult |
| 18 | 11454 | y |  |  | CF:Mild, Pseudomonas | Female | White | American | Child |
|  | 11455 |  | y |  | CF:Parental Control | Female | White | American | Adult |
| 19 | 11474 | y |  |  | CF:Mild, Staph | Male | White | American | Child |
|  | 11475 |  | y |  | CF:Parental Control | Female | White | American | Adult |
| 20 | 11634 | y |  |  | CF:Mild, Staph | Female | White | American | Child |
|  | 11635 |  | y |  | CF:Parental Control | Female | White | American | Adult |
| 21 | 11694 | y |  |  | CF:Mild, Staph | Female | White | American | Child |
|  | 11695 |  | y |  | CF:Parental Control | Female | White |  | Adult |
| 22 | 11104 | y |  |  | CF:Severe, Pseudomonas | Male | White | American | Adult |
|  | 11105 |  | y |  | CF:Parental Control | Female | White | American | Adult |
|  | 11106 |  |  | y | CF:Parental Control | Male | White | American | Adult |
| 23 | 11204 | y |  |  | CF:Mild, Pseudomonas | Female | White | American | Child |
|  | 11205 |  | y |  | CF:Parental Control | Female | White | American | Adult |
|  | 11206 |  |  | y | CF:Parental Control | Male | White | American | Adult |
| 24 | 11254 | y |  |  | CF:Moderate, Pseudomonas | Female | White | American | Child |
|  | 11255 |  | y |  | CF:Parental Control | Female | White | American | Adult |
|  | 11256 |  |  | y | CF:Parental Control | Male | White | American | Adult |
| 25 | 11324 | y |  |  | CF:Severe, Pseudomonas | Male | White | American | Child |
|  | 11325 |  | y |  | CF:Parental Control | Female | White | American | Adult |
| 26 | 11344 | y |  |  | CF:Mild, Pseudomonas | Male | White | American | Child |
|  | 11345 |  | y |  | CF:Parental Control | Female | White |  | Adult |
|  | 11346 |  |  | y | CF:Parental Control | Male | White | American | Adult |
| 27 | 11364 | y |  |  | CF:Mild, Staph | Male | White | American | Child |
|  | 11365 |  | y |  | CF:Parental Control | Female | White | American | Adult |
|  | 11366 |  |  | y | CF:Parental Control | Male | White | American | Adult |
| 28 | 11485 | y |  |  | CF:Mild, Pseudomonas | Female | White | American | Adult |
|  | 11487 |  | y |  | CF:Grandparent Control | Female | White | American | Adult |
| 29 | 11514 | y |  |  | CF:Mild, Staph | Male | White | American | Child |
|  | 11515 |  | y |  | CF:Parental Control | Female | White | American | Adult |
|  | 11516 |  |  | y | CF:Parental Control | Male | White | American | Adult |
| 30 | 11564 | y |  |  | CF:Mild, Staph | Male | White | American | Child |
|  | 11565 |  | y |  | CF:Parental Control | Female | White | American | Adult |
|  | 11566 |  |  | y | CF:Parental Control | Male | White | American | Adult |
| 31 | 11334 | y |  |  | CF:Mild, Staph | Male | White | American | Child |
|  | 11335 |  | y |  | CF:Parental Control | Female | White | American | Adult |
|  | 11336 |  |  | y | CF:Parental Control | Male | White | American | Adult |
| 32 | 11314 | y |  |  | CF:Mild, Staph | Male | White | American | Child |
|  | 11315 |  | y |  | CF:Parental Control | Female | White | American | Adult |
|  | 11316 |  |  | y | CF:Parental Control | Male | White | American | Adult |
|  | 11317 |  | y |  | CF:Grandparent Control | Female | White | American | Adult |
| 33 | 11504 | y |  |  | CF:Moderate, Pseudomonas | Female | White | American | Child |
|  | 11505 |  | y |  | CF:Parental Control | Female | White | American | Adult |
|  | 11506 |  |  | y | CF:Parental Control | Male | White | American | Adult |
| 34 | 11524 | y |  |  | CF:Mild, Pseudomonas | Male | White | American | Adult |
|  | 11525 |  | y |  | CF:Parental Control | Female | White | American | Adult |
|  | 11526 |  |  | y | CF:Parental Control | Male | White | American | Adult |
| 35 | 11574 | y |  |  | CF:Mild, Pseudomonas | Female | White | American | Child |
|  | 11575 |  | y |  | CF:Parental Control | Female | White | American | Adult |
|  | 11576 |  |  | y | CF:Parental Control | Male | White | American | Adult |
| 36 | 11583 | y |  |  | CF:Mild, Pseudomonas | Female | White | American | Child |
|  | 11584 | y |  |  | CF:Mild, Pseudomonas | Male | White | American | Child |
|  | 11585 |  | y |  | CF:Parental Control | Female | White | American | Adult |
|  | 11586 |  |  | y | CF:Parental Control | Male | White | American | Adult |
| 37 | 11614 | y |  |  | CF:Mild, Pseudomonas | Male | White | American | Child |
|  | 11615 |  | y |  | CF:Parental Control | Female | White | American | Adult |
|  | 11616 |  |  | y | CF:Parental Control | Male | White | American | Adult |
| 38 | 11664 | y |  |  | CF:Mild, Staph | Female | White | American | Child |
|  | 11665 |  | y |  | CF:Parental Control | Female | White | American | Adult |
| 39 | 11164 | y |  |  | CF:Severe, Pseudomonas | Female | White | American | Adult |
|  | 11165 |  | y |  | CF:Parental Control | Female | White | American | Adult |
|  | 11166 |  |  | y | CF:Parental Control | Male | White | American | Adult |
| 40 | 11374 | y |  |  | CF:Moderate, Pseudomonas | Male | White | American | Child |
|  | 11375 |  | y |  | CF:Parental Control | Female | White | American | Adult |
|  | 11376 |  |  | y | CF:Parental Control | Male | White | American | Adult |
| 41 | 11084 | y |  |  | CF:Mild, Pseudomonas | Female | Hispanic | Unknown | Child |
|  | 11085 |  | y |  | CF:Parental Control | Female | Hispanic | Mexican | Adult |
| 42 | 11194 | y |  |  | CF:Moderate, Pseudomonas | Female | White | American | Child |
|  | 11195 |  | y |  | CF:Parental Control | Female | White | American | Adult |
|  | 11196 |  |  | y | CF:Parental Control | Male | White | American | Adult |
| 43 | 11264 | y |  |  | CF:Mild, Staph | Female | White | American | Child |
|  | 11265 |  | y |  | CF:Parental Control | Female | White | American | Adult |
|  | 11268 |  |  | y | CF:Grandparent Control | Male | White | American | Adult |
| 44 | 11414 | y |  |  | CF:Mild, Pseudomonas | Male | White | American | Child |
|  | 11415 |  | y |  | CF:Parental Control | Female | White | American | Adult |
|  | 11416 |  |  | y | CF:Parental Control | Male | White | American | Adult |
| 45 | 11404 | y |  |  | CF:Mild, Pseudomonas | Female | White | American | Child |
|  | 11405 |  | y |  | CF:Parental Control | Female | White | American | Adult |
|  | 11406 |  |  | y | CF:Parental Control | Male | White | American | Adult |
| 46 | 11094 | y |  |  | CF:Mild, Pseudomonas | Male | White | American | Child |
|  | 11095 |  | y |  | CF:Parental Control | Female | White | American | Adult |
|  | 11096 |  |  | y | CF:Parental Control | Male | White | American | Adult |
| 47 | 11484 | y |  |  | CF:Mild, Pseudomonas | Female | White | American | Child |
|  | 11486 |  |  | y | CF:Parental Control | Male | White | American | Adult |
| 48 | 11554 | y |  |  | CF:Mild, Staph | Female | White | American | Child |
|  | 11555 |  | y |  | CF:Parental Control | Female | White | American | Adult |
|  | 11556 |  |  | y | CF:Parental Control | Male | White | American | Adult |
| 49 | 11824 | y |  |  | CF:Mild, Pseudomonas | Female | White | American | Child |
|  | 11823 | y |  |  | CF:Mild, Staph | Male | White | American | Child |
|  | 11825 |  | y |  | CF:Parental Control | Female | White | American | Adult |
|  | 11826 |  |  | y | CF:Parental Control | Male | White | American | Adult |
| 50 | 11703 | y |  |  | CF:Mild, Pseudomonas | Female | White | American | Child |
|  | 11704 | y |  |  | CF:Mild, Pseudomonas | Female | White | American | Child |
|  | 11705 |  | y |  | CF:Parental Control | Female | White | American | Adult |
|  | 11706 |  |  | y | CF:Parental Control | Male | White | American | Adult |
| 51 | 11434 | y |  |  | CF:Mild, Pseudomonas | Male | White | American | Child |
|  | 11435 |  | y |  | CF:Parental Control | Female | White | American | Adult |
|  | 11436 |  |  | y | CF:Parental Control | Male | White | American | Adult |
| 52 | 11644 | y |  |  | CF:Mild, Pseudomonas | Male | White | American | Child |
|  | 11645 |  | y |  | CF:Parental Control | Female | White | American | Adult |
|  | 11646 |  |  | y | CF:Parental Control | Male | White | American | Adult |
| 53 | 11224 | y |  |  | CF:Moderate, Pseudomonas | Male | White | American | Child |
|  | 11225 |  | y |  | CF:Parental Control | Female | White | American | Adult |
|  | 11226 |  |  | y | CF:Parental Control | Male | White | American | Adult |
| 54 | 11154 | y |  |  | CF:Moderate, Pseudomonas | Female | White | American | Adult |
|  | 11155 |  | y |  | CF:Parental Control | Female | White | American | Adult |
|  | 11157 |  | y |  | CF:Grandparent Control | Female | White | American | Adult |
| 55 | 11284 | y |  |  | CF:Moderate, Staph | Female | White | American | Child |
|  | 11285 |  | y |  | CF:Parental Control | Female | White | American | Adult |
|  | 11286 |  |  | y | CF:Parental Control | Male | White | American | Adult |
| 56 | 11354 | y |  |  | CF:Mild, Pseudomonas | Male | White | American | Child |
|  | 11355 |  | y |  | CF:Parental Control | Female | White | American | Adult |
|  | 11356 |  |  | y | CF:Parental Control | Male | White | American | Adult |
| 57 | 11674 | y |  |  | CF:Mild, Staph | Female | White | American | Child |
|  | 11676 |  |  | y | CF:Parental Control | Male | White | American | Adult |
|  | 11675 |  | y |  | CF:Parental Control | Female | White | American | Adult |
| 58 | 11754 | y |  |  | CF:Mild | Female | White | American | Child |
|  | 11755 |  | y |  | CF:Parental Control | Female | White | American | Adult |
|  | 11756 |  |  | y | CF:Parental Control | Male | White | American | Adult |
| 59 | 11683 | y |  |  | CF:Mild | Female | White | American | Child |
|  | 11684 | y |  |  | CF:Moderate, Staph | Female | White | American | Child |
|  | 11685 |  | y |  | CF:Parental Control | Female | White | American | Adult |
| 60 | 11794 | y |  |  | CF:Mild | Female | White | American | Child |
|  | 11795 |  | y |  | CF:Parental Control | Female | White | American | Adult |
|  | 11796 |  |  | y | CF:Parental Control | Male | White | American | Adult |
| 61 | 11834 | y |  |  | CF:Mild, Staph | Female | White | American | Child |
|  | 11835 |  | y |  | CF:Parental Control | Female | White | American | Adult |
|  | 11836 |  |  | y | CF:Parental Control | Male | White | American | Adult |
| 62 | 11854 | y |  |  | CF:Mild, Staph | Male | White | American | Child |
|  | 11855 |  | y |  | CF:Parental Control | Female | White | American | Adult |
|  | 11856 |  |  | y | CF:Parental Control | Male | White | American | Adult |
| 63 | 11814 | y |  |  | CF:Mild, Pseudomonas | Female | White | American | Adult |
|  | 11816 |  |  | y | CF:Parental Control | Male | White | American | Adult |
| 64 | 11724 | y |  |  | CF:Moderate, Other | Female | White | American | Adult |
|  | 11725 |  | y |  | CF:Parental Control | Female | White | American | Adult |
| 65 | 11764 | y |  |  | CF:Mild, Staph | Male | White | American | Child |
|  | 11765 |  | y |  | CF:Parental Control | Female | White | American | Adult |
| 66 | 11476 | y |  |  | CF:Mild | Male | White | American | Adult |
|  | 11479 |  | y |  | CF:Grandparent Control | Female | White | American | Adult |
|  | 11480 |  |  | y | CF:Grandparent Control | Male | White | American | Adult |
| 67 | 11784 | y |  |  | CF:Mild | Female | White | American | Child |
|  | 11785 |  | y |  | CF:Parental Control | Female | White | American | Adult |
|  | 11786 |  |  | y | CF:Parental Control | Male | White | American | Adult |
| 68 | 11534 | y |  |  | CF:Mild, Pseudomonas | Male | White | American | Adult |
|  | 11535 |  | y |  | CF:Parental Control | Female | White | American | Adult |
| 69 | 11874 | y |  |  | CF:Mild, Staph | Female | White | American | Child |
|  | 11875 |  | y |  | CF:Parental Control | Female | White | American | Adult |
|  | 11876 |  |  | y | CF:Parental Control | Male | White | American | Adult |
| 70 | 11494 | y |  |  | CF:Mild, Pseudomonas | Female | White | American | Child |
|  | 11493 | y |  |  | CF:Mild, Pseudomonas | Female | White | American | Child |
|  | 11495 |  | y |  | CF:Parental Control | Female | White | American | Adult |
|  | 11496 |  |  | y | CF:Parental Control | Male | White | American | Adult |
| 71 | 11071 |  |  |  | CF:Sibling Control | Female | White | American | Adult |
|  | 11074 | y |  |  | CF:Mild, Pseudomonas | Male | White | American | Child |
|  | 11075 |  | y |  | CF:Parental Control | Female | White | American | Adult |
|  | 11666 |  |  |  | CF:Parental Control | Male | White | American | Adult |
| 72 | 11024 | y |  |  | CF:Moderate, Pseudomonas | Female | White | American | Child |
|  | 11026 |  |  | y | CF:Parental Control | Male | White | American | Adult |
|  | 11025 |  | y |  | CF:Parental Control | Female | White | American | Adult |
|  | 11027 |  | y |  | CF:Grandparent Control | Female | White | American | Adult |
|  | 11028 |  |  | y | CF:Grandparent Control | Male | White | American | Adult |

1) A total of 72 pedigrees: 43 pedigrees where each has one case with 2 parents; 21 pedigrees where each has one case with one parent; 5 pedigrees where each has two cases in each family, 4 pedigrees where each has three generations.

2) Race: 198 are white, 7 are Hispanic. Nationality: 196-American, 6-Mexican, and 3- unknown.

3) CF disease: 79 cases, 4-severe, 11-moderate, and 64-mild.

y; yes
