# Supplementary material for: Genetic Association of Pulmonary Surfactant Protein Genes, SFTPA1, SFTPA2, SFTPB, SFTPC, and SFTPD With Cystic Fibrosis
Source: Front Immunol. 2018 Oct 2;9:2256. doi: 10.3389/fimmu.2018.02256 (PMC6175982; doi:10.3389/fimmu.2018.02256)
Supplement: Supplementary file 2 [file Table_2.docx]

**Supplementary Table 2.** Geno type frequencies in mild and moderate CF-cases compared to control.

| **Mild CF** | | | | | | | | | |
| --- | --- | --- | --- | --- | --- | --- | --- | --- | --- |
| **Gene** | **Variation ID** | | **Other used name** | | **Nucleotide** | | **Genotype Frequency** | | |
|  |  |  |  |  |  |  | **CF-case** | | **Control** |
| SFTPA2 | rs1059046 | | aa9 Asn/Thr | | AC/AA/CC | | 0.47/0.379/0.152 | | 0.51/0.316/0.173 |
|  | rs17886395 | | aa91 Pro/Ala | | GG/CG/CC | | 0.727/0.242/0.03 | | 0.735/0.235/0.031 |
|  | rs1965707 | | aa140 Ser/Ser | | CT/CC/TT | | 0.409/0.561/0.03 | | 0.408/0.52/0.071 |
|  | rs1965708 | | aa223 Gln/Lys | | CA/CC/AA | | 0.318/0.682/0 | | 0.316/0.643/0.041 |
| SFTPA1 | rs1059047 | | aa19 Ala/Val | | TT/CT/CC | | 0.833/0.152/0.015 | | 0.857/0.133/0.01 |
|  | rs1136450 | | aa50 Leu/Val | | CG/GG/CC | | 0.485/0.333/0.182 | | 0.551/0.296/0.153 |
|  | rs1136451 | | aa62 Pro/Pro | | GA/AA/GG | | 0.303/0.621/0.076 | | 0.286/0.684/0.031 |
|  | rs1059057 | | aa133 Thr/Thr | | GA/AA/GG | | 0.167/0.818/0.015 | | 0.122/0.867/0.01 |
|  | rs4253527 | | aa219 Arg/Trp | | CT/CC/TT | | 0.273/0.727/0 | | 0.163/0.816/0.02 |
| SFTPB | rs2077079 | | CA-18, CA1022 | | AA/CA/CC | | 0.348/0.53/0.121 | | 0.306/0.531/0.163 |
|  | rs3024798 | | CA1013, CA2052 | | CC/AC/AA | | 0.333/0.545/0.121 | | 0.347/0.541/0.112 |
|  | rs1130866 | | TC1580, TC2619  aa131 Ile/Thr | | TC/CC/TT | | 0.576/0.136/0.288 | | 0.5/0.194/0.306 |
|  | rs7316 | | AG9306, AG10345 | | AA/AG | | 0.818/0.182 | | 0.939/0.061 |
| SFTPC | rs4715* | | aa138 Asn/Thr | | AC | | 1 | | 1 |
|  | rs1124* | | aa186 Asn/Ser | | AG | | 1 | | 1 |
| SFTPD | rs721917 | | aa11 Met/Thr | | TC/CC/TT | | 0.485/0.167/0.348 | | 0.571/0.194/0.235 |
|  | rs2243639 | | aa160 Thr/Ala | | GG/AG/AA | | 0.364/0.409/0.227 | | 337/0.51/0.153 |
| **Moderate CF** | | | | | | | | | |
| **Gene** | **Variation ID** | **Other used name** | | **Nucleotide** | | **Genotype Frequency** | | | |
|  |  |  |  |  |  | **CF-case** | | **Control** | |
| SFTPA2 | rs1059046 | aa9 Asn/Thr | | AA/AC/CC | | 0.357/0.571/0.071 | | 0.318/0.409/0.273 | |
|  | rs17886395 | aa91 Pro/Ala | | GG/CG/CC | | 0.643/0.357/0 | | 0.682/0.273/0.045 | |
|  | rs1965707 | aa140 Ser/Ser | | CC/CT/TT | | 0.571/0.429/0 | | 0.636/0.273/0.091 | |
|  | rs1965708 | aa223 Gln/Lys | | CC/CA/AA | | 0.714/0.286/0 | | 0.727/0.227/0.045 | |
| SFTPA1 | rs1059047 | aa19 Ala/Val | | TT/CT/CC | | 0.786/0.214/0 | | 0.773/0.182/0.045 | |
|  | rs1136450 | aa50 Leu/Val | | GG/CG/CC | | 0.357/0.5/0.143 | | 0.227/0.5/0.273 | |
|  | rs1136451 | aa62 Pro/Pro | | AA/GA/GG | | 0.786/0.214/0 | | 0.636/0.318/0.045 | |
|  | rs1059057 | aa133 Thr/Thr | | AA/GA/GG | | 0.786/0.214/0 | | 0.773/0.182/0.045 | |
|  | rs4253527 | aa219 Arg/Trp | | CC/CT | | 0.929/0.071 | | 0.818/0.182 | |
| SFTPB | rs2077079 | CA-18, CA1022 | | AA/CA/CC | | 0.5/0.286/0.214 | | 0.5/0.364/0.136 | |
|  | rs3024798 | CA1013, CA2052 | | CC/AC/AA | | 0.429/0.357/0.214 | | 0.636/0.273/0.091 | |
|  | rs1130866 | TC1580, TC2619  aa131 Ile/Thr | | TC/TT/CC | | 0.571/0.286/0.143 | | 0.364/0.273/0.364 | |
|  | rs7316* | AG9306, AG10345 | | AG | | 1 | | 1 | |
| SFTPC | rs4715* | aa138 Asn/Thr | | AC | | 1 | | 1 | |
|  | rs1124* | aa186 Asn/Ser | | AG | | 1 | | 1 | |
| SFTPD | rs721917 | aa11 Met/Thr | | TT/TC/CC | | 0.214/0.786/0 | | 0.318/0.5/0.182 | |
|  | rs2243639 | aa160 Thr/Ala | | AA/AG/GG | | 0.286/0.5/0.214 | | 0.182/0.455/0.364 | |

*These SNP have only one genotype detected, and frequency is 1.
