# Supplementary material for: Genetic Association of Pulmonary Surfactant Protein Genes, SFTPA1, SFTPA2, SFTPB, SFTPC, and SFTPD With Cystic Fibrosis
Source: Front Immunol. 2018 Oct 2;9:2256. doi: 10.3389/fimmu.2018.02256 (PMC6175982; doi:10.3389/fimmu.2018.02256)
Supplement: Supplementary file 3 [file Table_3.docx]

**Supplementary Table 3.** Epistatic intragenic and intergenic interactions of SNPs in surfactant genes, SFTPA1, SFTPA2, SFTPB,

SFTPC, and SFTPD.

|  |  | SFTPA1 | | | | | SFTPA2 | | | | SFTPB | | | | SFTPC | | SFTPD | |
| --- | --- | --- | --- | --- | --- | --- | --- | --- | --- | --- | --- | --- | --- | --- | --- | --- | --- | --- |
|  |  | rs1059047 | rs1136450 | rs1136451 | rs1059057 | rs4253527 | rs1059046 | rs17886395 | rs1965707 | rs1965708 | rs2077079 | rs3024798 | rs1130866 | rs7316 | rs4715 | rs1124 | rs721917 | rs2243639 |
| SFTPA1 | rs1059047 |  |  |  |  |  |  |  |  |  |  |  |  |  | 0.0487 | 0.0053 |  |  |
|  | rs1136450 |  |  |  |  |  | 0.0048 |  |  |  |  |  |  | 0.0213 | 0.0182 | 0.0187 |  |  |
|  | rs1136451 |  |  |  | 0.0468 | 0.0238 |  |  |  |  |  |  |  |  | 0.0328 | 0.0007 |  |  |
|  | rs1059057 |  |  |  |  |  |  |  |  |  |  |  |  | 0.0332 |  | 0.0007 |  |  |
|  | rs4253527 |  |  |  |  |  | 0.0233 |  |  |  |  |  |  | 0.0303 |  | 0.0404 |  |  |
| SFTPA2 | rs1059046 |  |  |  |  |  |  |  |  |  |  |  | 0.039 |  | 0.0227 | 0.0471 |  |  |
|  | rs17886395 |  |  |  |  |  |  |  |  |  |  |  |  |  |  | 0.0038 |  |  |
|  | rs1965707 |  |  |  |  |  |  |  |  |  | 0.0043 |  |  |  |  |  |  |  |
|  | rs1965708 |  |  |  |  |  |  |  |  |  | 0.0262 | 0.0331 |  |  |  |  |  |  |
| SFTPB | rs2077079 |  |  |  |  |  |  |  |  |  |  | 0.0325 |  |  |  | 0.0344 | 0.0108 | 0.0058 |
|  | rs3024798 |  |  |  |  |  |  |  |  |  |  |  |  |  |  |  | 0.0398 | 0.0058 |
|  | rs1130866 |  |  |  |  |  |  |  |  |  |  |  |  |  | 0.0121 | 0.0207 | 0.0193 | 0.0382 |
|  | rs7316 |  |  |  |  |  |  |  |  |  |  |  |  |  |  | 0.0303 |  | 0.0094 |
| SFTPC | rs4715 |  |  |  |  |  |  |  |  |  |  |  |  |  |  |  | 0.0049 |  |
|  | rs1124 |  |  |  |  |  |  |  |  |  |  |  |  |  |  |  | 0.0075 | 0.0247 |
| SFTPD | rs721917 |  |  |  |  |  |  |  |  |  |  |  |  |  |  |  |  |  |
|  | rs2243639 |  |  |  |  |  |  |  |  |  |  |  |  |  |  |  |  |  |

p value is shown in the table. Total 37 interactions: 3 intragenic (highlight grey) and 34 intergenic.
