# Supplementary material for: Genetic Association of Pulmonary Surfactant Protein Genes, SFTPA1, SFTPA2, SFTPB, SFTPC, and SFTPD With Cystic Fibrosis
Source: Front Immunol. 2018 Oct 2;9:2256. doi: 10.3389/fimmu.2018.02256 (PMC6175982; doi:10.3389/fimmu.2018.02256)
Supplement: Supplementary file 4 [file Table_4.docx]

**Supplementary Table 4.** Surfactant protein genes with CF disease severity subgroups (mild and moderate/severe).

**Moderate/severe subgroup**

| 1 SNP model  p-Value <= 0.01:  No significant result  p-Value <= 0.05:  No significant result | | | | | | | | |
| --- | --- | --- | --- | --- | --- | --- | --- | --- |
| **Interaction #** | **SNP #1** | | **SNP #2** | | **Interaction type** | **x^2^** | **p value** | **Corrected p value** |
|  | **Gene name** | **SNP ID** | **Gene name** | **SNP ID** |  |  |  |  |
| **2 SNP model p-value <=0.01** | | | | | | | |  |
| 1 | SFTPA2 | rs1059046 | SFTPA1 | rs1136450 | a1 | 2.29978355 | 0.00205943 | 0.216239728 |
| 2 | SFTPA2 | rs1059046 | SFTPB | rs3024798 | a1d2 | 5.66504336 | 0.00792495 | 0.416059944 |
| 3 | SFTPA2 | rs17886395 | SFTPB | rs3024798 | d1d2 | 3.78081799 | 0.00577866 | 0.606759478 |
| 4 | SFTPA2 | rs1965708 | SFTPA1 | rs1136450 | d1d2 | 2.64286472 | 0.00890898 | 0.311814269 |
| 5 | SFTPA1 | rs1136450 | SFTPB | rs3024798 | a1d2 | 5.55555556 | 0.00382369 | 0.401487491 |
| 6 | SFTPB | rs3024798 | SFTPD | rs2243639 | d1d2 | 3.45982906 | 0.00828725 | 0.435080883 |
|  |  |  |  |  |  |  |  |  |
|  |  |  |  |  |  |  |  |  |
| **p-value <=0.05** |  |  |  |  |  |  |  |  |
| 1 | SFTPA2 | rs1059046 | SFTPA1 | rs4253527 | d1d2 | 2.46392552 | 0.02786835 | 0.418025183 |
| 2 | SFTPA2 | rs1059046 | SFTPB | rs2077079 | a1d2 | 3.83522727 | 0.02938139 | 0.514174252 |
| 3 | SFTPA2 | rs1059046 | SFTPB | rs1130866 | a1d2 | 4.62900776 | 0.01589005 | 0.5561519 |
| 4 | SFTPA2 | rs17886395 | SFTPB | rs3024798 | d1 | 2.56238943 | 0.02869572 | 1 |
| 5 | SFTPA2 | rs17886395 | SFTPB | rs3024798 | d2 | 2.56238943 | 0.03882891 | 1 |
| 6 | SFTPA2 | rs1965707 | SFTPA1 | rs1059047 | d1d2 | 2.53981245 | 0.02545989 | 0.445547995 |
| 7 | SFTPA2 | rs1965708 | SFTPD | rs721917 | d1d2 | 1.58601399 | 0.04655488 | 0.54314027 |
| 8 | SFTPA1 | rs1136450 | SFTPA1 | rs4253527 | a1 | 1.77777778 | 0.01041097 | 0.546576035 |
| 9 | SFTPA1 | rs1136450 | SFTPB | rs2077079 | a1d2 | 3.74523810 | 0.02219804 | 0.582698542 |
| 10 | SFTPA1 | rs1136450 | SFTPB | rs7316 | a1 | 1.16883117 | 0.03316653 | 0.870621372 |
| 11 | SFTPA1 | rs1136450 | SFTPD | rs2243639 | a1 | 2.21309524 | 0.01131277 | 0.395947012 |
| 12 | SFTPA1 | rs4253527 | SFTPD | rs721917 | d1d2 | 2.42944317 | 0.01473957 | 0.309531019 |
| 13 | SFTPB | rs3024798 | SFTPB | rs1130866 | d2 | 2.80000000 | 0.04547655 | 1 |
| 14 | SFTPB | rs3024798 | SFTPD | rs721917 | d1d2 | 1.60209059 | 0.03158550 | 0.414559691 |
| 15 | SFTPB | rs3024798 | SFTPD | rs2243639 | a1d2 | 3.01822059 | 0.02557721 | 0.537121493 |
| 16 | SFTPB | rs1130866 | SFTPD | rs721917 | d1d2 | 2.35874611 | 0.01381086 | 0.362535136 |
| 17 | SFTPD | rs721971 | SFTPD | rs2243639 | a1 | 1.26623377 | 0.04145124 | 0.870476079 |

**Mild group**

| p-Value <= 0.01:  1: SFTPB (rs7316) Dominance x2=10.38812785 0.00126828 corrected_pvalue: 0.01902422  p-Value <= 0.05:  1: SFTPA1 (rs4253527) Dominance x2=4.68981050 0.03034199 | | | | | | | | |
| --- | --- | --- | --- | --- | --- | --- | --- | --- |
| **Interaction #** | **SNP #1** | | **SNP #2** | | **Interaction type** | **x^2^** | **p value** | **Corrected p value** |
|  | **Gene name** | **SNP ID** | **Gene name** | **SNP ID** |  |  |  |  |
| **2 SNP model p-value <=0.01** | | | | | | | |  |
| 1 | SFTPA2 | rs1965708 | SFTPB | rs7316 | d2 | 3.66565180 | 0.00820001 | 0.430500750402981 |
| 2 | SFTPA1 | rs1136450 | SFTPB | rs7316 | d1d2 | 3.81926684 | 0.00607927 | 0.212774593242959 |
| 3 | SFTPA1 | rs1059057 | SFTPB | rs7316 | d2 | 4.77551020 | 0.00931902 | 0.326165669 |
| 4 | SFTPA1 | rs4253527 | SFTPB | rs7316 | d2 | 5.55638114 | 0.00510446 | 0.535968190998152 |
| 5 | SFTPB | rs3024798 | SFTPD | rs721917 | d1d2 | 5.47559633 | 0.00077503 | 0.0813784537377527 |
| 6 | SFTPB | rs1130866 | SFTPD | rs721917 | d1a2 | 6.90124062 | 0.00057516 | 0.0603920585643652 |
| 7 | SFTPB | rs1130866 | SFTPD | rs2243639 | d1a2 | 5.83841692 | 0.00299812 | 0.157401459533076 |
| 8 | SFTPB | rs7316 | SFTPD | rs2243639 | d1d2 | 3.49498711 | 0.00210045 | 0.110273506356421 |
|  |  |  |  |  |  |  |  |  |
| **p-value <=0.05** | | | | | | | | |
| 1 | SFTPA2 | rs1059046 | SFTPA2 | rs17886395 | a1a2 | 3.72748258 | 0.03691383 | 1 |
| 2 | SFTPA2 | rs1059046 | SFTPD | rs721917 | d1d2 | 2.64401193 | 0.03025412 | 0.453811865347012 |
| 3 | SFTPA2 | rs17886395 | SFTPD | rs721917 | d1 | 3.21744069 | 0.03199813 | 1 |
| 4 | SFTPA2 | rs1965707 | SFTPB | rs2077079 | d1d2 | 2.61683297 | 0.02323410 | 0.406596773212319 |
| 5 | SFTPA2 | rs1965707 | SFTPB | rs7316 | d2 | 3.66077084 | 0.02241018 | 0.336152712150163 |
| 6 | SFTPA2 | rs1965708 | SFTPA1 | rs4253527 | d2 | 2.09746310 | 0.04417576 | 0.386537921866667 |
| 7 | SFTPA2 | rs1965708 | SFTPB | rs2077079 | d1d2 | 2.15699236 | 0.03701464 | 0.485817092118653 |
| 8 | SFTPA1 | rs1059047 | SFTPA1 | rs1136451 | d1 | 3.36275904 | 0.02485639 | 1 |
| 9 | SFTPA1 | rs1059047 | SFTPB | rs7316 | d2 | 4.51467051 | 0.02140325 | 0.561835344873985 |
| 10 | SFTPA1 | rs136451 | SFTPA1 | rs1059057 | d2 | 3.66666667 | 0.02230482 | 0.468401187900924 |
| 11 | SFTPA1 | rs136451 | SFTPA1 | rs4253527 | d2 | 3.66666667 | 0.02230482 | 0.39033432325077 |
| 12 | SFTPA1 | rs136451 | SFTPB | rs1130866 | a1 | 4.23111111 | 0.04333660 | 1 |
| 13 | SFTPA1 | rs136451 | SFTPB | rs7316 | d2 | 3.53110941 | 0.02486314 | 0.326328754916784 |
| 14 | SFTPA1 | rs136451 | SFTPB | rs7316 | a1d2 | 3.03349332 | 0.04574872 | 0.960723028765294 |
| 15 | SFTPA1 | rs1059057 | SFTPB | rs7316 | a1d2 | 3.05029285 | 0.04514264 | 1 |
| 16 | SFTPA1 | rs4253527 | SFTPB | rs7316 | d1 | 3.06742974 | 0.04526235 | 1 |
| 17 | SFTPA1 | rs4253527 | SFTPB | rs7316 | a1d2 | 4.36488829 | 0.01625327 | 0.853296627866773 |
| 18 | SFTPA1 | rs4253527 | SFTPB | rs7316 | d1a2 | 3.06742974 | 0.04011948 | 1 |
| 19 | SFTPB | rs2077079 | SFTPD | rs721917 | a1a2 | 3.65323565 | 0.03897430 | 1 |
| 20 | SFTPB | rs2077079 | SFTPD | rs721917 | d1d2 | 3.37652452 | 0.01478510 | 0.310487003787867 |
| 21 | SFTPB | rs2077079 | SFTPD | Rs2243639 | a1d2 | 4.78113288 | 0.01385300 | 1 |
| 22 | SFTPB | Rs3024798 | SFTPB | rs7316 | d2 | 2.56210669 | 0.02650476 | 0.278299932597749 |
| 23 | SFTPB | Rs3024798 | SFTPD | Rs2243639 | d1d2 | 3.08078179 | 0.01335266 | 0.350507233949895 |
| 24 | SFTPB | Rs1130866 | SFTPD | Rs2243639 | d2 | 3.70084699 | 0.02584995 | 0.301582731878977 |
| 25 | SFTPB | rs7316 | SFTPD | rs721917 | d1 | 2.60351811 | 0.04871556 | 1 |
| 26 | SFTPB | rs7316 | SFTPD | Rs2243639 | d2 | 2.46955362 | 0.02931407 | 0.279816147572322 |
| 27 | SFTPB | rs7316 | SFTPD | Rs2243639 | a1d2 | 2.46955362 | 0.03865746 | 1 |
| Note: SNP marked with yellow color denotes significant association after Bonferroni correction. | | | | | | | | |
